# Dimeric Lectin Chimeras as Novel Candidates for Gb3-Mediated Transcytotic Drug Delivery through Cellular Barriers

**DOI:** 10.3390/pharmaceutics15010225

**Published:** 2023-01-09

**Authors:** Maokai Xu, Maria Antonova, Pavel Salavei, Katharina Illek, Ana Valeria Meléndez, Ramin Omidvar, Roland Thuenauer, Olga Makshakova, Winfried Römer

**Affiliations:** 1 Faculty of Biology, University of Freiburg, 79104 Freiburg, Germany; 2 Signaling Research Centers BIOSS and CIBSS, University of Freiburg, 79104 Freiburg, Germany; 3 Kazan Institute for Biochemistry and Biophysics, FRC Kazan Scientific Center of RAS, 420111 Kazan, Russia; 4 Spemann Graduate School of Biology and Medicine (SGBM), University of Freiburg, 79104 Freiburg, Germany; 5 Center for Structural Systems Biology (CSSB), 22607 Hamburg, Germany; 6 Technology Platform Light Microscopy, University of Hamburg, 20146 Hamburg, Germany; 7 Technology Platform Microscopy and Image Analysis (TPMIA), Leibniz Institute of Virology (LIV), 20251 Hamburg, Germany; 8 Freiburg Institute for Advanced Studies (FRIAS), University of Freiburg, 79104 Freiburg, Germany

**Keywords:** Gb3-binding lectin, glycosphingolipid, transcytosis, drug carrier, protein engineering, protein structure, valency, accelerated molecular dynamics

## Abstract

Receptor-mediated transcytosis is an elegant and promising strategy for drug delivery across biological barriers. Here, we describe a novel ligand–receptor pair based on a dimeric, engineered derivative of the *Pseudomonas aeruginosa* lectin LecA, here termed Di-LecA, and the host cell glycosphingolipid Gb3. We characterized the trafficking kinetics and transcytosis efficiencies in polarized Gb3-positive and -negative MDCK cells using mainly immunofluorescence in combination with confocal microscopy. To evaluate the delivery capacity of dimeric LecA chimeras, EGFP was chosen as a fluorescent model protein representing macromolecules, such as antibody fragments, and fused to either the N- or C-terminus of monomeric LecA using recombinant DNA technology. Both LecA/EGFP fusion proteins crossed cellular monolayers in vitro. Of note, the conjugate with EGFP at the N-terminus of LecA (EGFP-LecA) showed a higher release rate than the conjugate with EGFP at the C-terminus (LecA-EGFP). Based on molecular dynamics simulations and cross-linking studies of giant unilamellar vesicles, we speculate that EGFP-LecA tends to be a dimer while LecA-EGFP forms a tetramer. Overall, we confidently propose the dimeric LecA chimeras as transcytotic drug delivery tools through Gb3-positive cellular barriers for future in vivo tests.

## 1. Introduction

Currently, the efforts in the field of drug delivery are concentrating on the delivery of biologics across cellular barriers and the targeting of pathological tissues or organs, such as the gastrointestinal tract, the central nervous system, and solid tumor tissues [1,2,3,4,5]. Thereby, cellular barriers in the human body affect drug bio-distribution and pharmacokinetics, leading in many cases to a drastically reduced efficiency of therapeutics. To avoid the high risks induced by injecting therapeutic material directly into target organs, one elegant and promising therapeutic strategy is to hijack natural transport pathways to increase tissue-specific targeting efficiency. A strategy known as receptor-mediated transcytosis (RMT) has been widely studied and utilized in the field of drug delivery [6]. Through binding to specific receptors, the ligands are taken up into the cellular cytoplasm, transported, and released after the ligand-containing vesicles reach the opposite plasma membrane (PM). Notably, RMT is specific, saturable, and highly regulated by many adaptor proteins and GTPases [7,8,9]. So far, several receptors undergoing RMT after ligand binding, such as the transferrin receptor, the insulin receptor, and the lipoprotein receptor, have been investigated and reviewed [10,11].

Recently, host cell glycosphingolipids have attracted considerable attention as targeted receptor molecules [12,13,14]. Several of them were reported to undergo transcytosis after being engaged by their ligands [15,16,17]. For instance, the bacterial lectins LecA from *Pseudomonas aeruginosa* and the B-subunit of Shiga toxin (StxB) from *Shigella dysenteriae* bound specifically to the glycosphingolipid Gb3 and transcytosed from the apical to the basolateral plasma membrane of Gb3-positive MDCK cells at around 30 min, and vice versa [18]. Of note, a fraction of StxB proceeded on the retrograde transport pathway to the Golgi apparatus, whereas LecA was elusive on this pathway. Applying lectin/GSL-driven transcytosis brings several advantages: (1) Lectins have variable structures with diverse specificities toward glycosylated host cell receptors. (2) The number and position of the carbohydrate-binding sites can be varied [19,20]. (3) Cells, which selectively express Gb3, provide a solid basis for increasing the targeting efficiency.

Based on our initial transcytosis studies [18] with the homo-pentameric StxB and the homo-tetrameric LecA (from now on referred to as Tetra-LecA), we wanted to address the impact of lectin valency and structure on transcytosis kinetics and efficiency by using a dimeric LecA homolog (from now on referred to as Di-LecA). It was obtained by inserting a 6×His-tag at the N-terminus of the Tetra-LecA at the gene level [21]. The His-tag impeded the regular assembly to a tetramer, leading to a dimeric structure with probably altered binding properties. In this study, we characterized the trafficking kinetics and transcytosis properties of Di-LecA in comparison to Tetra-LecA. We revealed that Di-LecA underwent faster transcytosis and reached higher release efficiency than Tetra-LecA. To prove whether a dimeric LecA chimera can deliver biologics across cellular monolayers, we chose enhanced green fluorescent protein (EGFP), with a molecular weight of ~27 kDa, as a model protein to fuse with LecA considering its larger molecule size that cannot cross the cell monolayer alone and because of the intrinsic fluorescent signal that can be easily tracked by microscopy. EGFP was fused with LecA either at the N-terminus or C-terminus of LecA in terms of the different assembly structures. Interestingly, the fusion protein in which EGFP was fused to the N-terminus of LecA (referred to as EGFP-LecA) performed considerably faster trafficking kinetics than the fusion protein in which EGFP was fused to the C-terminus of LecA (referred to as LecA-EGFP). In addition, much less cross-linking of neighboring cells was observed with EGFP-LecA. Molecular dynamics simulations of monomeric LecA with varying linker positions helped to better understand its assembly in oligomeric structures and interpret the different binding and trafficking properties. The probability of LecA-EGFP forming a tetramer with two binding pockets oriented in one direction and two facing the opposite direction is higher, while EGFP-LecA might rather form a dimer with the two binding pockets facing the same direction. As a major finding of our study, we illustrate the potential of dimeric Gb3-binding LecA chimeras to be used as a drug delivery tool in transcellular drug delivery. Meanwhile, our results open up the possibility of developing LecA chimeras in vivo for increasing drug efficiency in the future.

## 2. Materials and Methods

### 2.1. Cell Culture 

Madin–Darby canine kidney cells (MDCK II cells), kindly provided by Prof. Enrique Rodriguez-Boulan (Weill Cornell Medical College, New York, NY, USA), were chosen as a cell model system to construct a polarized, tight cell monolayer in vitro. Given the fact that wild-type MDCK cells (WT-MDCK cells) do not naturally express Gb3, a Gb3-synthase and G418-resistance encoding plasmid were transfected into the WT-MDCK cells to obtain Gb3-expressing MDCK cells (henceforth called Gb3-positive MDCK cells) [18]. WT-MDCK cells and Gb3-positive MDCK cells were cultivated in Dulbecco’s modified Eagle’s medium (DMEM) (Life Technologies GmbH, Darmstadt, Germany) supplemented with 5% fetal calf serum (FCS) (Life Technologies GmbH, Darmstadt, Germany) and 2 mM L-glutamine (7Bioscience GmbH, Freiburg im Breisgau, Germany). To maintain the Gb3 expression in MDCK cells, 1 mg/mL G418 (Carl Roth GmbH, Karlsruhe, Germany) was added to the cell culture medium. In addition, human colorectal adenocarcinoma Caco-2 cells (HTB-37, American Type Culture Collection, Manassas, VA, USA) were used as an additional cell model for the trafficking kinetics studies. Caco-2 cells grew in DMEM supplemented with 20% FCS and 2 mM L-glutamine. All cells were cultivated at 37 °C in a humid atmosphere with 5% CO_2_.

### 2.2. Lectin Labeling

Tetra-LecA and Di-LecA were labeled separately with the fluorescent dye Alexa Fluor 488 (AF488) (Life Technologies GmbH, Darmstadt, Germany) by an N-hydroxysuccinimide (NHS) ester reaction. Briefly, lectins mixed with a 10-fold excess of dye were incubated for 1 h at room temperature (RT). Zeba Spin desalting columns (Life Technologies GmbH, Darmstadt, Germany) were used to purify the labeled lectins and remove the excess non-labeled dye. For double-labeled lectin-containing biotin and the fluorescent dye, the lectin was first incubated with a 5-fold excess of biotin-PEG_4_-NHS (Life Technologies GmbH, Darmstadt, Germany) for 1 h at RT, then purified with the Zeba Spin desalting column, and ultimately mixed with AF488 for another 1 h of incubation at RT.

### 2.3. Immunofluorescence Microscopy

WT-MDCK cells and/or Gb3-positive MDCK cells were seeded on glass coverslips (3 × 10^4^ cells) and incubated overnight to allow the cells to adhere. The next day, the cells were treated with 100 nM fluorescently labeled Tetra-LecA or Di-LecA for the indicated time periods. A 4% paraformaldehyde (PFA) (Carl Roth GmbH, Karlsruhe, Germany) solution was used to fix cells for 15 min at RT. To stop fixation and saturate free aldehyde groups, cells were incubated with a 50 mM NH_4_Cl (Carl Roth GmbH, Karlsruhe, Germany) solution for 5 min. Cell membranes were permeabilized with a so-called SAPO solution that contained 0.02% (*w/v*) saponin (Sigma-Aldrich GmbH, Taufkirchen, Germany) and 0.2% (*w/v*) BSA (Carl Roth GmbH, Karlsruhe, Germany) in DPBS^-/-^ (Life Technologies GmbH, Darmstadt, Germany). Subsequently, cells were stained with primary antibodies toward the target proteins overnight at 4 °C. After removing non-bound antibodies, cells were incubated with corresponding fluorescently labeled secondary antibodies and counterstained with 4′,6-diamidine-2-phenylindole (DAPI) (Carl Roth GmbH, Karlsruhe, Germany). Samples were imaged by means of a laser scanning confocal microscope (Nikon A1R equipped with a 60x/1.42NA oil objective and four laser lines at 405 nm, 488 nm, 561 nm, and 640 nm). Image analysis was performed using NIS-Elements 5.20.01 (Nikon) and Image J 1.52 V. A maximum intensity projection was chosen to present the acquisitions. The Mander’s co-localization coefficient M1 was calculated using the Coloc-2 plugin in Fiji Image J 1.0.

### 2.4. Pulse-Chase Assay

WT-MDCK cells and/or Gb3-positive MDKC cells (3 × 10^5^ cells) were seeded on Transwell filters and cultivated for 4–5 days until the tight junctions (TJs) were well developed, which was proven by measuring the trans-epithelial electrical resistance (TEER) as described in [18]. After the polarization of epithelial cells, lectins were added to the apical (Ap) or basolateral (Bl) PM by applying ice-cold cell culture medium supplemented with 100 nM Tetra-LecA-biotin-AF488 or Di-LecA-biotin-AF488 for 30 min at 4 °C (‘pulse phase’). The excess of unbound lectins was removed by two washing steps with ice-cold culture medium. Cellular trafficking processes were enabled by changing to a heated cell-culture medium (37 °C). Different incubation periods (0 min, 30 min, 60 min, 120 min, and 240 min) at 37 °C were carried out in a humidified cell culture incubator (‘chase phase’). To stop transcytosis after the chase phase, cells were instantly moved out from the incubator and cooled down in ice-cold culture medium. To detect the transcytosed lectins, 200 µL of ice-cold culture medium supplemented with 2 µg/mL streptavidin-AF647 (Life Technologies GmbH, Darmstadt, Germany) was applied for 30 min at 4 °C to the chamber opposite to the one in which the lectins were initially incubated. The chamber where streptavidin-AF647 was applied was washed twice with ice-cold cell culture medium. Afterward, cells were washed with ice-cold DPBS^-/-^ before fixing with 4% PFA for 10 min at 4 °C and additionally for 10 min at RT. To stop fixation, cells were incubated with a 50 mM NH_4_Cl buffer for 5 min and three washing steps with DPBS^-/-^ followed. Cells were permeabilized for 30 min at RT by a SAPO solution that was supplemented with DAPI to stain the nuclei.

The samples were analyzed with the NIS-Elements 5.20.01 software (Nikon Europe B.V., Amstelveen, The Netherlands). To enable a significant comparison of the measured signal intensities within each channel, the acquisition and quantification settings were kept similar for all time points and all repetitions of the experiments. The signal intensities of different channels were quantified with MATLAB 9.2 software [18]. The surface-to-surface transcytosis efficiency was calculated by the intensity of the red streptavidin signal (‘transcytosed lectin’) compared to the green lectin signal (‘total lectin’).

### 2.5. Release Efficiency Measurements (Medium-to-Medium Transcytosis)

Gb3-positive MDCK cells were seeded on Transwell filters for 4–5 days. Tetra-LecA and/or Di-LecA were labeled with the fluorescent dye AF488, and the fluorescence intensity of 200 nM Tetra-LecA-AF488 and/or Di-LecA-AF488 in the culture medium was thereafter measured by a microplate reader. The read-out value was set as I, and the average value was calculated according to $(\sum_{i}^{n}I)/n$). The fluorescence intensity of the cell culture medium (*I_medium_*) was set as a background value. After different incubation periods, 200 µL of the medium from both compartments was taken out and added to a 96-well plate to measure the fluorescence intensity (values are marked as *I_Bl_* or *I_Ap_,* indicating either Bl or Ap compartment intensity, respectively). Taking Tetra-LecA treatment from the Ap-to-Bl direction as an example: ($(\sum_{i}^{n}I)/n$ − *I_Ap_*) represents the total internalization quantity of Tetra-LecA, and (*I_Bl_* − *I_medium_*) is the transcytosed amount of Tetra-LecA. According to the following formula, the release efficiency can be calculated:

(1) $$P=\frac{I_{Bl}-I_{medium}}{(\sum_{i}^{n}I)/n-I_{Ap}}\times 100\%$$

### 2.6. Production, Purification, and Validation of Fusion Proteins

The fusion proteins (EGFP-LecA and LecA-EGFP) were produced in *E. coli* NiCo21 (DE3) (New England Biolabs GmbH, Frankfurt am Main, Germany) after the transformation of the plasmids pET-28a(+)-EGFP-LecA and pET-28a(+)-LecA-EGFP, respectively. The ÄKTA prime plus chromatography system (GE Healthcare, Leverkusen, Germany) was used for the first round of protein purification. A HisTrap FF Crude column (Cytiva, Freiburg im Breisgau, Germany), which uses an Ni-NTA agarose stationary phase, was utilized in this system. After purification, the fusion protein samples were transferred into a SnakeSkin dialysis tube (Life Technologies GmbH, Darmstadt, Germany) with a rated molecule weight cut-off of 10 kDa and dialyzed overnight with ice-cold DPBS^-/-^. The next day, the dialysis buffer was renewed, and samples were continually dialyzed for another 5 h. Then, the fusion protein samples were collected and prepared for size exclusion chromatography (SEC) as a second round of purification. Gel filtration chromatography with DPBS^-/-^ as a buffer was used as a polishing step for fusion protein purification. Fractions eluted from the affinity column containing EGFP-LecA and LecA-EGFP were pooled together and additionally purified on a HiLoad 26/600 Superdex 200 pg column (Cytiva, Freiburg im Breisgau, Germany) using an ÄKTA avant system (Cytiva, Freiburg im Breisgau, Germany).

### 2.7. Molecular Dynamics Simulations

The initial structures of LecA and EGFP were taken from PDB (code: 5D21 and 1EMA, respectively). Based on these structures, fusion proteins were built up using Modeller9.15 [22]. The molecular systems used for simulations are listed in the Supplementary Materials (Figure S11).

Accelerated molecular dynamics (AMD) was carried out to effectively decrease the energy barriers and accelerate conformational transitions [23]. More detailed information can be found in the Supplementary Materials. The analysis of the trajectories was performed using the STRIDE module implemented in VMD software [24]. Dihedral angle principal component analysis (DPCA) and cluster analysis were performed using the Carma program [25]. Further analysis was performed using in-house python scripts.

### 2.8. Statistical Analysis

Statistical analysis was performed with GraphPad Prism8 (version 8.4.3). Statistical parameters are presented in figures and figure legends, including the individual sample numbers (*n*), precision measures (mean or mean ± SEM), and statistical significance. Data were judged to be statistically significant with a *p*-value smaller than 0.05. In figures, asterisks denote statistical significance, which was calculated either by the Mann–Whitney test or unpaired Student’s *t*-test (* *p* < 0.05; ** *p* < 0.01; **** *p* < 0.0001; ns, not significant).

## 3. Results

### 3.1. Di-LecA Undergoes Faster Transcytosis Than Tetra-LecA

**Figure 1:**
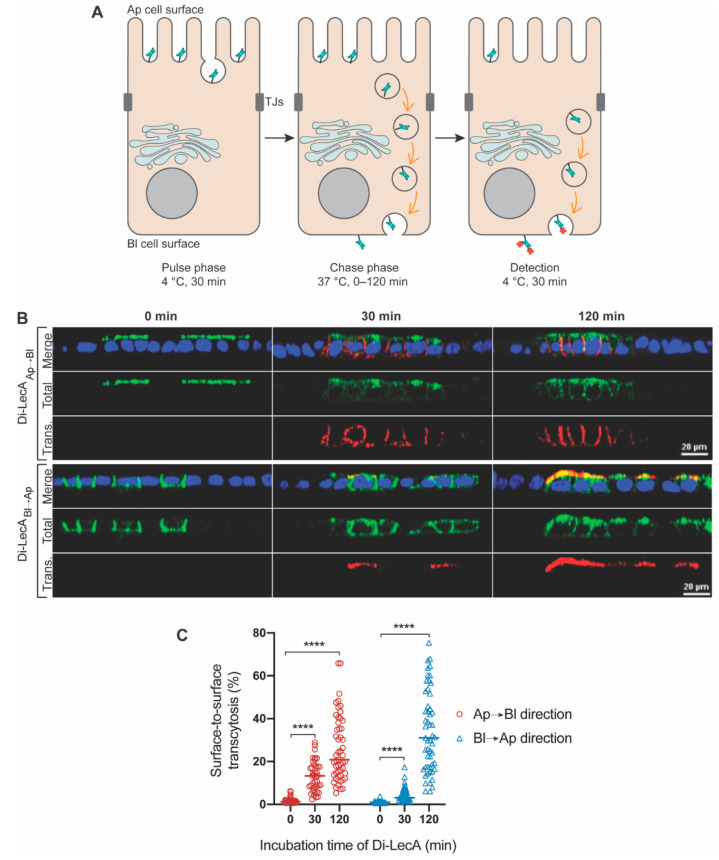
Surface-to-surface transcytosis of Di-LecA. (**A**) A schematic illustration of the applied ‘Pulse-Chase’ assay. The biotinylated lectin was, in addition, labeled with the fluorophore AF488, which is shown in green color in the scheme. Di-LecA-biotin-AF488 was pre-bound to the Ap or the Bl cell surface for 30 min at 4 °C. Excess Di-LecA-biotin-AF488 was removed by washing with ice-cold medium. Cells were shifted to 37 °C for the indicated incubation periods: 0 min, 30 min, and 120 min, respectively. After the incubation, cells were cooled to 4 °C, and Di-LecA arrival was detected by using streptavidin-AF647, shown in red color in the scheme. (**B**) Transcytosed Di-LecA-biotin-AF488 (green) was measured. Representative cross-sections of acquired z-stack images are shown. Cell-surface-bound Di-LecA-biotin-AF488 was detected by using streptavidin-AF647 (red) from the opposite side (scale bar: 20 μm). (**C**) The surface-to-surface transcytosis efficiencies of Di-LecA were quantified. The Mann–Whitney test was used to calculate the significant difference between groups. Mean values are shown. **** *p* < 0.0001. For each time point, the included individual cell number *n* was greater or equal to 30.

The last step of transcytosis is the contact and fusion of the transported vesicles with the PM. The quantity of ligands at the cellular surface after transcytosis theoretically reflects the transport efficiency. To characterize the trafficking kinetics and the surface-to-surface transcytosis efficiency of Di-LecA, we performed a Pulse-Chase assay. For the assay, cellular monolayers composed of Gb3-positive MDCK cells were used on Transwell filters, as established in [18]. For better comparison, biotinylated lectins (Di-LecA or Tetra-LecA; 100 nM), which were additionally labeled with AF488 (in green color) were separately applied either to the Ap or the Bl cell surface for 30 min at 4 °C (‘Pulse phase’). After the completion of the Pulse phase, the cells were shifted to 37 °C for up to 120 min (‘Chase phase’) for lectin internalization into the cells. Ultimately, the cells were moved back to 4 °C to stop the internalization and trafficking processes, and streptavidin-AF647 (in red color) was used to detect the arrival of biotinylated lectins at the cell surface (schematics in Figure 1A). Notably, neither Di-LecA nor Tetra-LecA were observed to bind to Gb3-negative WT-MDCK cells during 30 min of incubation at 4 °C (Figure S1).

Compared to the weak signal of Tetra-LecA, which was observed after 30 min of internalization (Figure S2A), a much more pronounced signal of Di-LecA appeared after 30 min of transport from the Ap to Bl direction (Figure 1B). An enhanced signal of Di-LecA was observed after 120 min in both directions of transcytosis (Figure 1B). To investigate the surface-to-surface transcytosis efficiency, we quantified the ratios using the arrival signal intensity divided by the integrated lectin signal intensity (illustrated in Section 2.4). The quantifications showed that around 12% of the Di-LecA accumulated after 30 min at the Bl surface when applied before to the Ap surface, and 20% after 120 min (Figure 1C). Moreover, around 5% (after 30 min) and 30% (after 120 min) of Di-LecA were detected at the Ap surface after transcytosis from Bl to the Ap cell surface (Figure 1C).

Compared to Tetra-LecA, for which around 10% (at Bl surface) and 15% (at Ap surface), respectively, were observed after 120 min (Figure S2B), the transcytosed quantity of Di-LecA was significantly higher (Figure S2C). A two-fold higher efficiency ratio of Di-LecA compared to Tetra-LecA could be obtained in both directions of transcytosis. Thus, we concluded that Di-LecA performed faster transcytosis than Tetra-LecA in our cell model.

### 3.2. Di-LecA Mainly Bypasses the Trans-Golgi Network 

Most molecules enter early endosomes after their uptake into target cells, from where they can, e.g., either return to the PM via recycling endosomes or enter the degradative pathway via late endosomes to lysosomes [26]. However, some molecules escape from recycling or degradation and enter other compartments, such as the Golgi apparatus and the endoplasmic reticulum (ER) [27]. For instance, Shiga toxin from *Shigella dysenteriae or* enterohaemorrhagic *Escherichia coli (EHEC)*, considered a representative cargo of the retrograde transport route, is transported from the PM to the ER by passing through the Golgi apparatus/trans-Golgi network [28]. Our previous published study that analyzed the transcytotic trafficking of Gb3-binding lectins [18] illustrated that recycling endosomes are an essential central hub for Tetra-LecA trafficking in epithelial cells. However, it is unknown so far if Di-LecA has similar trafficking properties.

Thus, we performed initial immunofluorescence experiments to verify whether Di-LecA enters recycling endosomes after its cellular uptake into non-polarized cells. The fluorescently labeled Di-LecA (in green color) co-localized with the recycling endosome marker Rab11 (in red color) at 30 min of incubation at 37 °C (Figure S3A). Interestingly, Di-LecA accumulated in the perinuclear area, whereas Tetra-LecA was still more homogeneously distributed at this time point (Figure S3A,B). To characterize the trafficking properties of Di-LecA in more detail in polarized cells, we grew cellular monolayers composed of Gb3-positive MDCK cells on Transwell filters and performed immunofluorescence experiments in combination with confocal microscopy by using the TGN marker TGN46 and the lysosome marker Lamp1. As before, we performed the assay in both the Ap to Bl and Bl to Ap directions [29].

**Figure 2:**
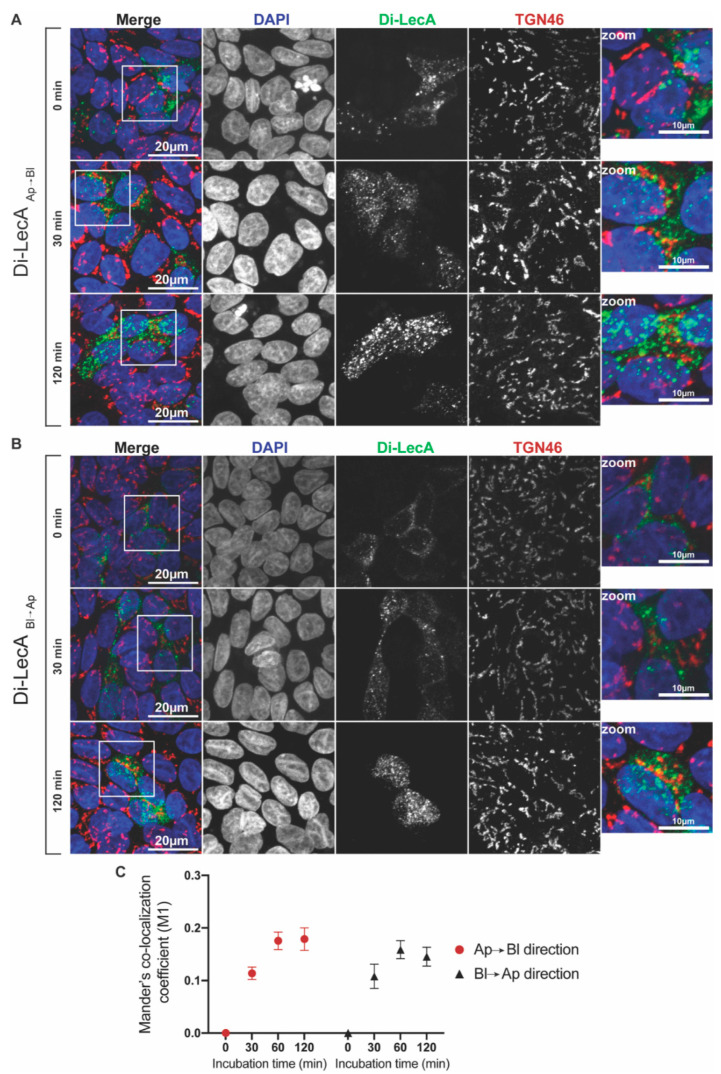
Co-localization analysis of Di-LecA with the trans-Golgi network marker TGN46. (**A**) Fluorescent Di-LecA (green) was pre-bound to the Ap cell surface of Gb3-positive MDCK cells at 4 °C for 30 min. After washing away unbound lectin, cells were shifted to 37 °C for up to 120 min to allow endocytosis and intracellular trafficking from Ap to the Bl side. After fixation and permeabilization, nuclei were stained with DAPI (blue), and TGN46 was stained with a primary antibody, followed by incubation with the corresponding secondary antibody (red). Images were acquired via confocal microscopy, and representative maximum intensity projections are presented (scale bar: 20 µm; scale bar in the zoom: 10 µm). (**B**) Fluorescent Di-LecA was pre-bound to the Bl cell surface and intracellular trafficking from Bl to the Ap side through the TGN was analyzed. Precisely the same experimental conditions were applied as described in (**A**). (**C**) Mander’s M1 co-localization coefficient between Di-LecA and TGN46 was calculated via the Coloc-2 plugin in ImageJ for different time points (0–120 min) of transport in the Ap→Bl and Bl→Ap directions. For each time point, the included individual cell number *n* was greater or equal to 30. The error bars represent the SD.

Our immunofluorescence studies already showed qualitatively that only a little Di-LecA (in green) co-localized with TGN46 (in red) after 30 min up to 120 min of transport in both directions (Figure 2A,B). To further analyze the co-localization, we quantified Mander’s co-localization coefficient M1. For both directions of trafficking, the co-localization coefficient between Di-LecA and TGN46 was determined at around 0.1 after 30 min, suggesting very low co-localization (Figure 2C). It increased up to 0.2 at 60 min of trafficking and also remained at this plateau level at 120 min (Figure 2C). We concluded from these studies that the Di-LecA mainly bypasses the TGN and that only a minor fraction undergoes retrograde transport. Similar results have also been determined for Tetra-LecA (Figure S4). Additionally, trafficking studies of Di-LecA and Tetra-LecA followed by co-localization analysis have been performed in polarized Caco-2 cells. All co-localization coefficients of Di-LecA with TGN46 were below 0.1, even smaller than the values determined in Gb3-positive MDKC cells (Figure S5).

### 3.3. Small Quantities of Di-LecA Arrive in Lysosomes 

For a suitable drug carrier, as little loss of material as possible is desirable. Lysosomes are the major degradative compartments in eukaryotic cells. Proteases and enzymes in lysosomes can digest a wide variety of structurally diverse substances [30,31]. Therefore, we determined whether Di-LecA can enter the degradative trafficking pathways. We selected the Lysosomal-associated membrane protein 1 (Lamp1) as the marker to track the distribution of lysosomes in cells [32]. By imaging the co-localization of fluorescent Di-LecA and Lamp1 via confocal microscopy, we were able to verify whether the lectin was directed toward lysosomes in its intracellular trafficking pathways.

**Figure 3:**
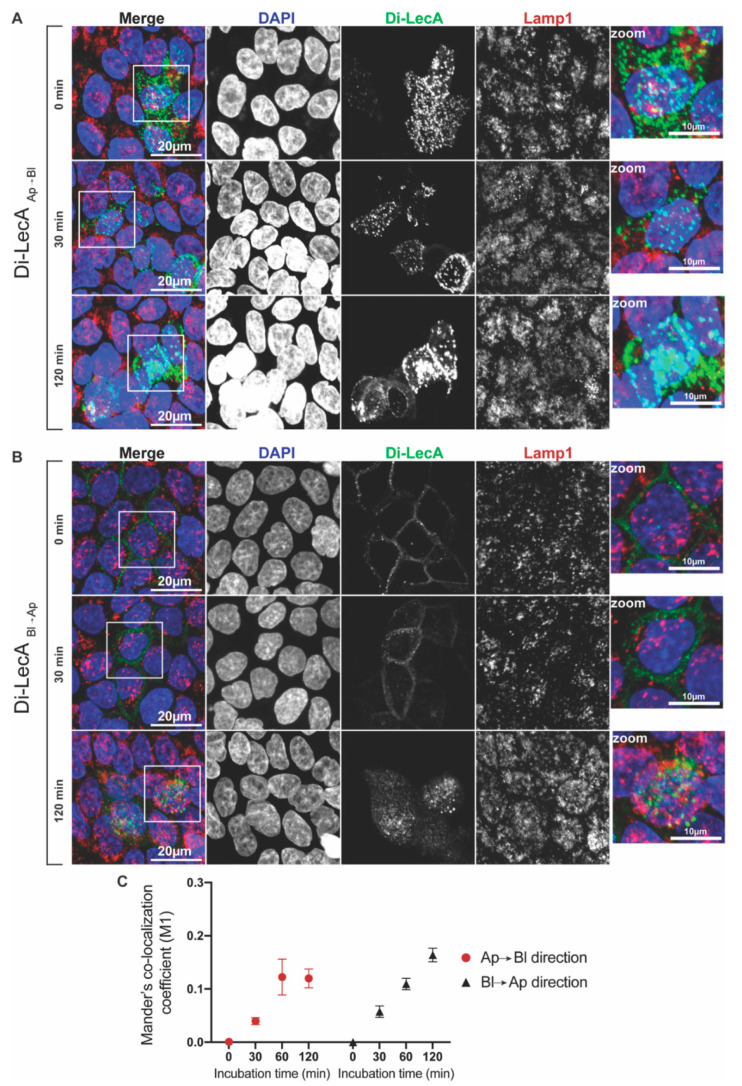
Co-localization analysis of Di-LecA with lysosomes. (**A**) Fluorescent Di-LecA (green) was pre-bound to the Ap cell surface of Gb3-positive MDCK cells at 4 °C for 30 min. After removing the excess unbound lectin, cells were shifted to 37 °C, allowing trafficking processes from the Ap to Bl membrane. After the fixation and permeabilization of cells, nuclei were stained with DAPI (blue), and Lamp1 was stained with a primary antibody, followed by incubation with the corresponding secondary antibody (red). Images were acquired via confocal microscopy, and representative maximum intensity projections are presented (scale bar: 20 µm; scale bar in the zoom: 10 µm). (**B**) Fluorescent Di-LecA was pre-bound to the Bl cell surface and intracellular trafficking from Bl to the Ap side through lysosomes was analyzed. Similar experimental conditions were applied as described in (**A**). (**C**) Mander’s M1 co-localization coefficient between Di-LecA and Lamp1 was calculated via the Coloc-2 plugin in ImageJ for different time points (0–120 min) of transport in the Ap→Bl and Bl→Ap directions. For each time point, the included individual cell number *n* was greater or equal to 30. The error bars represent the SD.

Immunofluorescence studies showed that a negligible quantity of Di-LecA accumulated in the Lamp1-associated compartments in both transport directions after 30 min and 120 min (Figure 3A,B). The degree of overlap was quantified by using Mander’s co-localization coefficient M1. The values were around 0.05 for 30 min of Ap-to-Bl transport and reached 0.12 for 60 min and 120 min (Figure 3C). Remarkably, the values of Bl-to-Ap trafficking were around 0.05 for 30 min and peaked at 0.18 for 60 to 120 min (Figure 3C).

Di-LecA showed similar transport properties to Tetra-LecA in Gb3-positive MDCK cells, i.e., both lectins mainly bypassed lysosomes (Figure S6). In Caco-2 cells, all determined co-localization coefficients of Di-LecA and Lamp1 were below 0.1 (Figure S7), lower than the values acquired in the Gb3-positive MDCK cells, again suggesting different trafficking performances in different cell lines. Collectively, the degree of overlap between Di-LecA and Lamp1 was low, suggesting that the degradation of both lectins in lysosomes was not a major issue in the used cell lines.

### 3.4. Di-LecA Is Slightly Better Released from Its Receptor Than Tetra-LecA

The most important parameter for evaluating the efficiency of transcellular delivery is the release of the ligand from its receptor after transcytosis. The release efficiency correlates strongly with the binding affinity/avidity of the ligand to its receptor. A high binding affinity can cause difficulties in detachment, leading to a low release ratio [33]. To characterize the release efficiency of Di-LecA and Tetra-LecA after transcytosis, we used the so-called ‘medium-to-medium’ transcytosis assay. As before, cellular monolayers were established on Transwell filters using Gb3-positive MDCK cells and WT-MDCK cells, respectively. Dextran-FITC with a molecular size of 20 kDa was used to confirm the impenetrability of the used cell monolayers in a paracellular way. Fluorescently labeled Di-LecA or Tetra-LecA was applied to either the top or the bottom compartment of the Transwell chambers. After their cellular uptake and partial transcytosis, the cell culture medium of both Transwell compartments was collected, and the fluorescence signal intensities of the corresponding lectins were measured using a microplate reader. The calculation of release efficiency followed the formula shown in Section 2.5.

**Figure 4:**
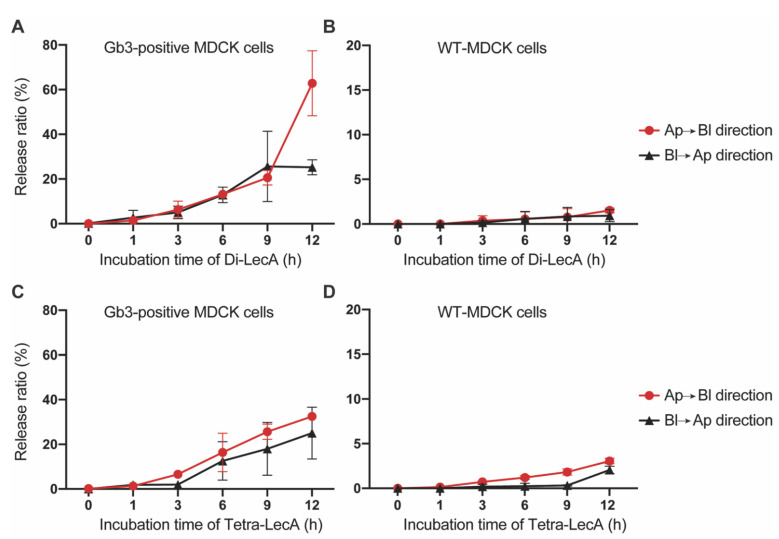
Release efficiencies of Di-LecA and Tetra-LecA. (**A**) The release efficiencies of Di-LecA were determined after crossing cellular monolayers that consist either of Gb3-positive MDCK cells (**A**) or Gb3-negative WT-MDCK cells (**B**). Fluorescently labeled Di-LecA was dropped in the top Transwell chamber or the bottom Transwell chamber. After the indicated incubation periods, fluorescent Di-LecA solution was collected from the chambers. The fluorescence intensity of the lectin solutions was measured via a microplate reader. The release efficiency was calculated according to the formula shown in Section 2.5. (**C**) The release efficiencies of Tetra-LecA were measured after crossing cellular monolayers consisting of either Gb3-positive MDCK cells (**C**) or Gb3-negative WT-MDCK cells (**D**). Monolayers that consist of WT-MDCK cells were used to confirm the specificity of lectin-triggered transcytosis. For each time point, the included sample number *n* was greater or equal to 3. Error bars represent the SD.

No dextran-FITC signal was detected in the opposite chamber until 12 h of incubation (Figure S8), suggesting that the TJs were well established and that no paracellular pathways existed in the surrogate Gb3-positive barrier model. In the transcytosis assays in the Ap to Bl direction, up to 60% of the Di-LecA penetrated the cell monolayer and was released after 12 h, whereas only 2% was detected after crossing the cell monolayer composed of Gb3-negative WT-MDCK cells (Figure 4A,B). Moreover, around 20% of Di-LecA was released in the culture medium after transcytosis in the Bl to Ap direction through the cell monolayer composed of Gb3-positive MDCK cells, and only around 2% was detected in the same direction of transcytosis through a cell monolayer consisting of WT-MDCK cells (Figure 4A,B). Regarding the release efficiency of Tetra-LecA, we can state that after transcytosis through Gb3-positive MDCK cell monolayers, around 30% of Tetra-LecA was detected after 12 h in the Bl culture medium when applying it from the Ap surface. Around 20% was detected in the Ap medium when applying it from the Bl surface (Figure 4C), while values for the release efficiency were at around 2.5% for Gb3-negative WT-MDCK cells (Figure 4D).

### 3.5. Transcytosis of Model LecA Fusion Proteins

**Figure 5:**
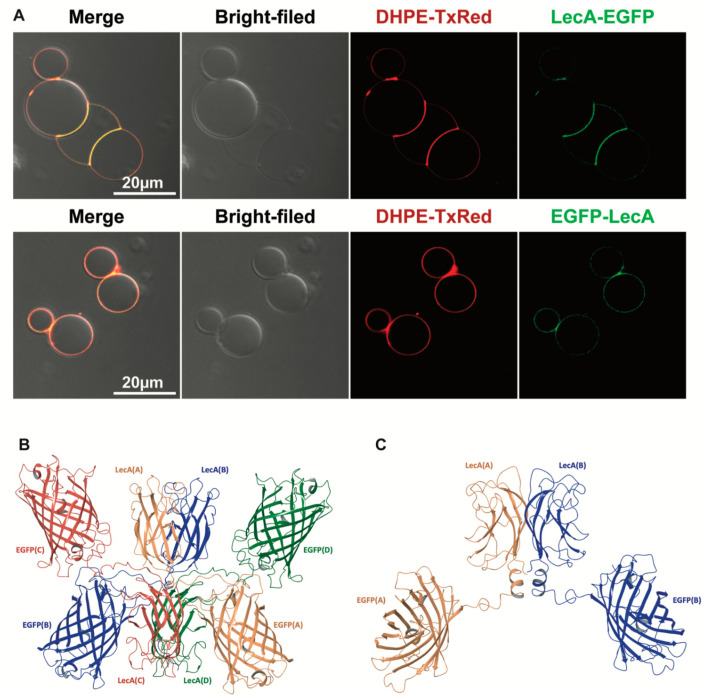
The properties of LecA-fusion proteins. (**A**) LecA-fusion proteins were used to test whether the fusion with EGFP at the N- or C-terminus can block the binding pockets of LecA to the glycosphingolipid Gb3. Gb3-containing GUVs (with Texas Red-labeled 1,2-dihexadecanoyl-sn-glycerol-3-phosphoethanolamine (DHPE-TR) as a fluorescent membrane marker) were separately incubated with LecA-EGFP and EGFP-LecA for 15 min at 37 °C (scale bar: 20 µm). (**B**) The putative structure of LecA-EGFP tetramer fusion protein in a cartoon with each chimeric subunit given in different colors. (**C**) The putative structure of EGFP-LecA dimer fusion protein in a cartoon with secondary structures given in different colors.

To show that Gb3-binding lectins can carry macromolecules across cellular barriers, we chose EGFP linked to LecA as a model cargo to prove our hypothesis. Keeping in mind the post-translational assembly mechanism of LecA, EGFP was fused with LecA at the *n*-terminus and C-terminus, respectively (Figure S9A). We produced the LecA-fusion proteins (LecA-EGFP and EGFP-LecA, respectively) in *E. coli* Nico21(DE3) cells and purified them using SEC (Figure S9B,C). These two conjugates were characterized by SDS-PAGE and Western blotting, and we observed that they possessed different molecular sizes in the gel: EGFP-LecA with a size of 39 kDa is smaller than LecA-EGFP with a size of 45 kDa (Figure S9D–F). The reason might be that the different spatial structures of these two fusion proteins slightly affect their migration speed in the SDS-PAGE gel. To determine whether EGFP negatively interferes with the carbohydrate-binding sites of LecA, we used Gb3-containing giant unilamellar vesicles (GUVs). This synthetic model system allowed us to study exclusively the interactions between the glycosphingolipid Gb3 as a receptor and LecA-fusion proteins as ligands. LecA-fusion proteins (200 nM) were applied to Gb3-containing GUVs that were enriched with the fluorescent lipid DHPE-TexasRed as a membrane marker (Figure 5A). A clear co-localization was observed between the fusion proteins and Gb3-containing GUVs after 15 min of incubation, suggesting that EGFP, either at the C- or N-terminus, obviously did not interfere with LecA binding to Gb3 (Figure 5A).

Moreover, we found that Gb3-containing GUVs were heavily cross-linked by LecA-EGFP, which became enriched at the interfaces of GUVs but much less by EGFP-LecA. Thus, at this point, we speculated that LecA-EGFP probably exists as a tetrameric protein that contains, as the natural homotetrameric LecA, opposite orientations of any two of its binding pockets, which then can cross-link GUVs as shown in Villringer et al. [34]. As EGFP-LecA did not clearly cross-link GUVs, we assumed a dimeric structure. 

We performed molecular dynamics simulations to obtain more insights into the chimeric LecA-based fusion protein structures and dynamics. An accelerated molecular dynamics (AMD) approach has been used to enhance the sampling. Such acceleration allowed for tackling protein folding, including helical [35] and beta structure formation [36,37,38]. In the framework of the current study, we hypothesized that the LecA fusion monomer would have altered N- and C-terminal structure and dynamics, which in turn would destabilize the A/C (B/D) contacts (Figure S10). As a result, the probability of the LecA tetramer formation would vary. Two molecular systems close to the experimental fusion constructs were considered (Figure S11A,B): a monomer of LecA (1) containing the linker sequence GGGS followed by the flag-tag sequence DYKDDDDK (from now on referred to as the ‘connecting sequence’) at the C-terminus, and (2) bearing the connecting sequence at the N-terminal and a 6×His-tag at the C-terminal end. In addition, the second construct was further diversified by two sequences where LecA bore only the connecting sequence or only the 6×His-tag to infer their cumulative effect. 

The analysis of the evolution of the connecting sequence in the course of AMD trajectories revealed its tendency to form a helix. The probability of the connecting sequence forming a helix was 25% when it was attached at the C-terminus. The probability reached 50% when the connecting sequence was located at the N-terminus of LecA. Furthermore, the simultaneous presence of a 6×His-tag at the C-terminus gave an additional stabilization of the connecting sequence as a rigid helix at the N-terminus. Here, the probability increased up to 65% (Figure S12). This stabilization occurs due to the interactions between the connecting sequence and the 6×His-tag. The increase in conformational rigidity in the fragment ‘Connecting sequence-LecA-6×His-tag’ can reduce the probability of the formation of a native-like tetramer due to the disruption of contacts in the A/C (B/D) dimers. Extrapolating this to LecA/EGFP fusion proteins, a rigid connecting sequence located at the N-terminus of LecA can result in a dimeric fusion protein where two LecA molecules form a dimer of the A/B (C/D) type. A less rigid connecting sequence located at the C-terminus of LecA appears to have less influence on the organization of the tetrameric fusion protein. This assessment is in line with the above-mentioned experimental findings that LecA-EGFP cross-linked two membrane surfaces, but EGFP-LecA binding to the membrane did not result in cross-linking.

Building up the whole structure of the fusion proteins by using the most probable structure of the connecting sequence and the 6×His-tag, we propose a possible model of the tetrameric LecA-EGFP fusion protein in Figure 5B, and a model of an A/B type dimer shown in Figure 5C for EGFP-LecA.

**Figure 6:**
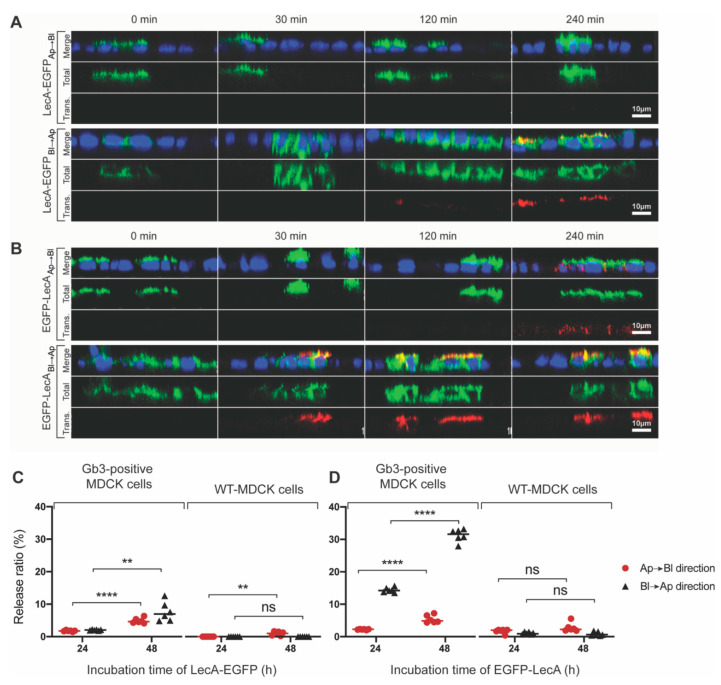
Transcytosis of LecA-fusion proteins. (**A**) Transcytosis of LecA-EGFP (green) in Gb3-positive MDCK cells illustrated by immunofluorescence. Firstly, LecA-EGFP was added either to the Ap or Bl cell surface and incubated at 4°C for 30 min. Then, cells were shifted to 37 °C for up to 240 min without further washing steps. Afterward, a primary antibody toward LecA was incubated at 4 °C to detect LecA-EGFP on the opposite side. A secondary antibody with AF647 (red) was used to detect the primary antibody toward LecA. (**B**) The transcytosis of EGFP-LecA (green) in Gb3-positive MDCK cells was illustrated by immunofluorescence. EGFP-LecA was pre-bound to the Ap or Bl cell surface at 4 °C for 30 min. Afterward, cells were incubated at 37 °C for the indicated time points without further washing steps. A primary antibody toward LecA was used at 4 °C for the detection of EGFP-LecA on the opposite side. A secondary antibody with AF647 (red) was used to detect the primary antibody of LecA. (**A**,**B**) Representative cross-sections of acquired z-stack images are shown (scale bar: 10 μm). (**C**,**D**) The release ratios of LecA-EGFP (**C**) and EGFP-LecA (**D**) were measured after 24 and 48 h of medium-to-medium transcytosis based on EGFP fluorescence by using a microplate reader. The release ratios were measured from two monolayers composed of either Gb3-positive MDCK cells or WT-MDCK cells. The unpaired Student’s *t*-test was used to calculate the significant differences between groups. Mean values are shown. ** *p* < 0.01; **** *p* < 0.0001; ns, not significant. For each time point, the included sample number *n* was equal to 6.

Furthermore, we conducted Pulse-Chase assays to investigate whether LecA-fusion proteins can cross cellular barriers consisting of polarized Gb3-positive MDCK cells. On Transwell filters, LecA-fusion proteins (presented in green color) were applied either to the Ap or the Bl cell surface. The Chase phases were set as 0 min, 30 min, 120 min, and 240 min. The arrival of the fusion proteins at the opposite cell surface was detected by an antibody against LecA (presented in red color). As illustrated in Figure 6A, no antibody signal was detected after 240 min of LecA-EGFP trafficking from Ap to the Bl side despite satisfying binding and internalization. However, in the opposite direction, from Bl to the Ap side, a small amount of LecA-EGFP was detected after 120 min, and an increased amount was detected after 240 min of trafficking. The experiments with the EGFP-LecA fusion construct were conducted in exactly the same manner. After trafficking from Ap to the Bl side, EGFP-LecA could be detected after 240 min (Figure 6B). Strikingly, EGFP-LecA was already detected after 30 min of trafficking from Bl to the Ap side (Figure 6B). It is obvious that the trafficking kinetics were superior for both constructs in the Bl to Ap direction. Here, EGFP-LecA performs best, probably due to its dimeric nature compared to the tetrameric LecA-EGFP with carbohydrate-binding sites oriented in the opposite direction. Nonetheless, the trafficking kinetics of LecA-fusion proteins seem to be slower than for corresponding Di- or Tetra-LecA.

We subsequently wanted to measure the release efficiencies of LecA-fusion proteins (medium-to-medium transcytosis) in order to determine whether or not these constructs were released into the cell culture medium after transcytosis. For this, we collected the cell culture medium from the Transwell chambers after 24 and 48 h of incubation and measured the EGFP fluorescence intensity of the medium using a microplate reader. Around 2.5% of LecA-EGFP was detected after 24 h of transport through polarized Gb3-positive MDCK cells in both directions (Figure 6C). By increasing the incubation time to 48 h, the release ratio increased to 5% (or more) in both directions (Figure 6C). Moreover, EGFP-LecA showed similar release efficiencies (2.5% and 5%, respectively) to LecA-EGFP after 24 and 48 h of trafficking from Ap to the Bl side (Figure 6D). However, in the opposite direction of trafficking, from Bl to the Ap side, around 15% of EGFP-LecA was released after 24 h and 30% after 48 h (Figure 6D).

In comparison, only negligibly small release values were measured after the incubation of Gb3-negative WT-MDCK cells with LecA-fusion constructs (Figure 6C,D). In particular, the release efficiencies from two directions of trafficking were observed to be similar at different time points, either for LecA-EGFP or for Tetra-LecA. Moreover, the release efficiencies of EGFP-LecA from Bl to the Ap side were higher than that from Ap to the Bl side after 24 h and 48 h. However, for Di-LecA, the release efficiency from Ap to the Bl side after 12 h was much higher than that in the opposite direction. It seems that a dimer of LecA has a more complicated trafficking mechanism compared to a tetramer.

## 4. Discussion

In the present study, we characterized the trafficking kinetics and transcytosis efficiency of the engineered dimeric LecA (Di-LecA) and LecA chimeras, which mainly hijack the glycosphingolipid Gb3 for transcytotic transport through polarized cell monolayers.

The concept of using bacterial lectins/toxins as delivery tools has been proposed previously [28,39,40]. For instance, the B-subunit of cholera toxin (CTB) was reported to be used in targeting gastric diseases and glioma, respectively, by recognizing the glycosphingolipid monosialotetrahexosylganglioside (GM1), which is i.a. abundant on the apical membrane of intestinal epithelial cells or brain endothelial cells [41,42,43,44]. The cholera toxin entered polarized cells via the retrograde trafficking route, and only a fraction of the toxin underwent transcytosis. Therefore, the usage of CTB focused mainly on intracellular drug delivery [2,45,46]. Strikingly, as only GM1 with short or unsaturated fatty acyl chains (i.e., C12:0-GM1 and C16:1-GM1, respectively) underwent transport from the apical to the basolateral membrane [47], the fatty acyl chain of GM1 has a strong impact on the endocytosis/transcytosis of CTB and accordingly on drug delivery [47]. The B-subunit of Shiga toxin (StxB), which binds to the glycosphingolipid Gb3 as a host cell receptor, has been studied as a drug carrier for a long time as well, specifically for targeting colorectal diseases [48,49]. Remarkably, Gb3-binding lectin-CAR T cells have demonstrated target-specific cytotoxicity against several cancer cell lines in vitro [50]. In addition, a bispecific construct composed of a Gb3-binding lectin linked to an antibody fragment (anti-CD3), termed ‘lectibody’, has been reported to kill Gb3-positive carcinoma cells in a highly specific and efficient manner by redirecting cytotoxic T lymphocytes in vitro [51]. Another Gb3-binding lectin recently suggested for therapeutical approaches, including transcytotic drug delivery, is the lectin LecA from *Pseudomonas aeruginosa* [18]. Even though StxB and LecA share Gb3 as their main receptor, they are segregated into different plasma membrane domains and trafficked along different transport pathways [18], which might probably be dependent on the length and degree of the saturation of the fatty acyl chain of Gb3. In a recent study, Schubert et al. pointed out differences in the binding preferences of StxB and LecA to diverse Gb3 species by using synthetic membrane systems [29]. In cell-based studies, Brandel et al. also highlighted differences in lectin preference for Gb3, which were dependent on the fatty acyl chain [52].

By exploiting their different binding preferences and trafficking pathways, it is intriguing to suggest the application of Gb3-binding lectins, in particular dimeric LecA-based chimeras, for the transcytotic delivery of drugs for targeting Alzheimer’s disease, brain carcinoma, or colon-related diseases, considering that Gb3 is highly abundant in the vascular endothelial cells of the brain and colon [53]. Di-LecA did not show much co-localization with the TGN, and only small portions entered lysosomes, suggesting low levels of retrograde transport and degradation. In polarized MDCK cells, we observed that Di-LecA showed faster trafficking kinetics and higher release efficiencies than Tetra-LecA. This could be explained by either the smaller molecular weight, and hence the facilitated transport of Di-LecA compared to Tetra-LecA, or the differences in structure and valency. Di-LecA possesses two carbohydrate-binding pockets oriented to the same side in contrast to Tetra-LecA, which comprises four carbohydrate-binding pockets with each two of the pockets facing opposite directions (Figure S10). In this sense, larger quantities of Tetra-LecA could remain caught at the plasma membrane at the interfaces of two neighboring cells, tightly cross-linking these cells. This cross-linking behavior of Tetra-LecA has already been observed by using cells and synthetic membrane systems [18,34]. On solid-supported lipid bilayers, Tetra-LecA formed membrane multilayers, which did not form by incubation with Di-LecA, probably due to the structural differences [54]. Hence, as Di-LecA lacks this cross-linking ability, its trafficking rates seem to be superior to Tetra-LecA (Figure 1 and Figure S2). Furthermore, valency could also play an important role in binding to the plasma membrane and subsequent endocytosis and transcytosis. For instance, Arnaud et al. revealed that the reduction in the valency of the *Ralstonia solanacearum* lectin (RSL) from six to three carbohydrate-binding sites did not really change its avidity to glycoconjugates but led to the disappearance of lectin-induced membrane invaginations on giant unilamellar vesicles and largely delayed its internalization into the human lung epithelial cell line H1299 in comparison to the native lectin [19]. To further elucidate the role of valency on avidity and host cell processes, monomeric neo-RSL mutants were designed with a controlled number of carbohydrate-binding sites (from six to zero) and different combinations of orientations to each other by engineering at the genomic level and mutating the gene sequences referring to the carbohydrate-binding sites [20]. At least two carbohydrate-binding sites were necessary to achieve similar avidities to that of native RSL to the receptor molecule. Moreover, the ability to bend and invaginate membranes was critically dependent on the distance between two adjacent carbohydrate-binding sites [20].

Additionally, we designed two LecA/EGFP-fusion proteins to identify whether LecA can deliver macromolecules at the size of antibody fragments (i.e., single-chain variable fragments) across cellular barriers. We first proved that the insertion of EGFP did not disturb LecA binding sites to Gb3 by conducting binding studies with GUVs. Here, we observed that LecA-EGFP induced the cross-linking of GUVs, whereas EGFP-LecA did not. This implies that LecA-EGFP binding sites to Gb3 are exposed in the opposite direction, like in the LecA tetramer, whilst the EGFP-LecA binding sites to Gb3 are exposed in the same direction. The latter may happen when the tetrameric organization of LecA is distorted by the insertion of EGFP at the N-terminus with a resultant dimer formation. This is in line with the following observation; namely, in the process of crossing cellular monolayers, EGFP-LecA underwent faster transcytosis than LecA-EGFP, suggesting that these two fusion proteins exhibit different trafficking properties. From the structural point of view, the weakest contacts stabilizing the LecA tetramer are those formed by the termini of the LecA subunit. The modification of the N-terminus by adding a 6×His-tag disturbs the tetrameric LecA organization [21,37]. Our simulations revealed that when the connecting sequence linking LecA and EGFP is at the N-terminus of LecA, it forms rigid secondary structures. The probability of this is confidently higher compared to when it is at the C-terminus. Combining the experimental findings with the molecular dynamics simulation studies, we propose that LecA-EGFP tends to be a tetramer while EGFP-LecA tends to be a dimer.

## 5. Conclusions

In the framework of this study, we aimed at shedding light on using engineered dimeric LecA as a drug delivery carrier to cross cellular barriers. We clarified that both natural (Tetra-LecA) and engineered LecA (Di-LecA) can penetrate the surrogate of cell monolayers with considerable efficiencies by binding to glycosphingolipid Gb3. Moreover, we first revealed that additive macromolecules conjugated to LecA such as EGFP did not interfere with the binding pockets to Gb3. Therefore, coupling LecA with established therapeutics such as chemical molecules, nucleic acids, and therapeutic antibodies is a promising strategy for increasing drug-targeting efficiencies [55,56,57,58]. In all, our findings demonstrate that lectins have excellent potential as drug delivery tools regarding their various structures, binding affinity, valency, specificity, and immunogenicity [59,60].

## Data Availability

Not applicable.

## Supplementary Materials

The following supporting information can be downloaded at: https://www.mdpi.com/article/10.3390/pharmaceutics15010225/s1. References cited in Supplementary Materials are [23,35,37,61,62,63,64,65,66,67,68,69,70].

## Abbreviations

RMT: receptor-mediated transcytosis; Di-LecA: dimeric LecA; Tetra-LecA: tetrameric LecA; EGFP-LecA: LecA-fusion protein that EGFP inserted at N-terminus; LecA-EGFP: LecA-fusion protein that EGFP inserted at C-terminus; h: hour(s); PM: plasma membrane; Pa: *Pseudomonas aeruginosa*; RT: room temperature; min: minute(s); TGN: the trans-Golgi network; ER: endoplasmic reticulum; Lamp1: lysosome-associated membrane protein 1; DAPI: 4′,6-diamidino-2-phenylindol.
