# Supplementary material for: Dimeric Lectin Chimeras as Novel Candidates for Gb3-Mediated Transcytotic Drug Delivery through Cellular Barriers
Source: Pharmaceutics. 2023 Jan 9;15(1):225. doi: 10.3390/pharmaceutics15010225 (PMC9864468; doi:10.3390/pharmaceutics15010225)
Supplement: Supplementary file 1 [file pharmaceutics-15-00225-s001.zip › pharmaceutics-2113010-supplementary.pdf]

Supplemental Materials

Supplemental Methods

Molecular Dynamics simulations

Molecular Dynamics (MD) simulations were performed starting from the initial structures immersed in water box with periodic boundary conditions. Simulations were carried out in Amber12 package [61] in the isotherm isobar thermodynamic ensemble at 300 K, using the Amber force-field parameters. The box was solvated and sodium and chloride ions were added in the amount necessary to neutralize the protein charge and to maintain the ionic strength of 150 mM. TIP3P model was used for water molecules. The integration step of 2 fs was used together with SHAKE algorithm constraining the bonds involving hydrogen atoms [62]. Particle mesh Ewald (PME) method was used for long-ranged electrostatic interactions [63]. A classical non-bond cut-off of 8 Å was chosen. The temperature and the pressure were kept constant using a Langevin thermostat with a collision frequency of 2 ps−1 and a weak coupling algorithm with a relaxation time of 2 ps−1, respectively. First, all systems were minimized for 5000 steps. In the production run of conventional MD, 200 ns were accumulated for each molecular system to get the potential energy and dihedral angles energy. These parameters were used to calculate the relative boost factors applied in the course of accelerated MD (AMD) [23]. The AMD technique effectively decreases the energy barriers and accelerates transitions, therefore was used to enhance conformational sampling. An around one microsecond AMD for each molecular system was accumulated.

Supplemental Figures

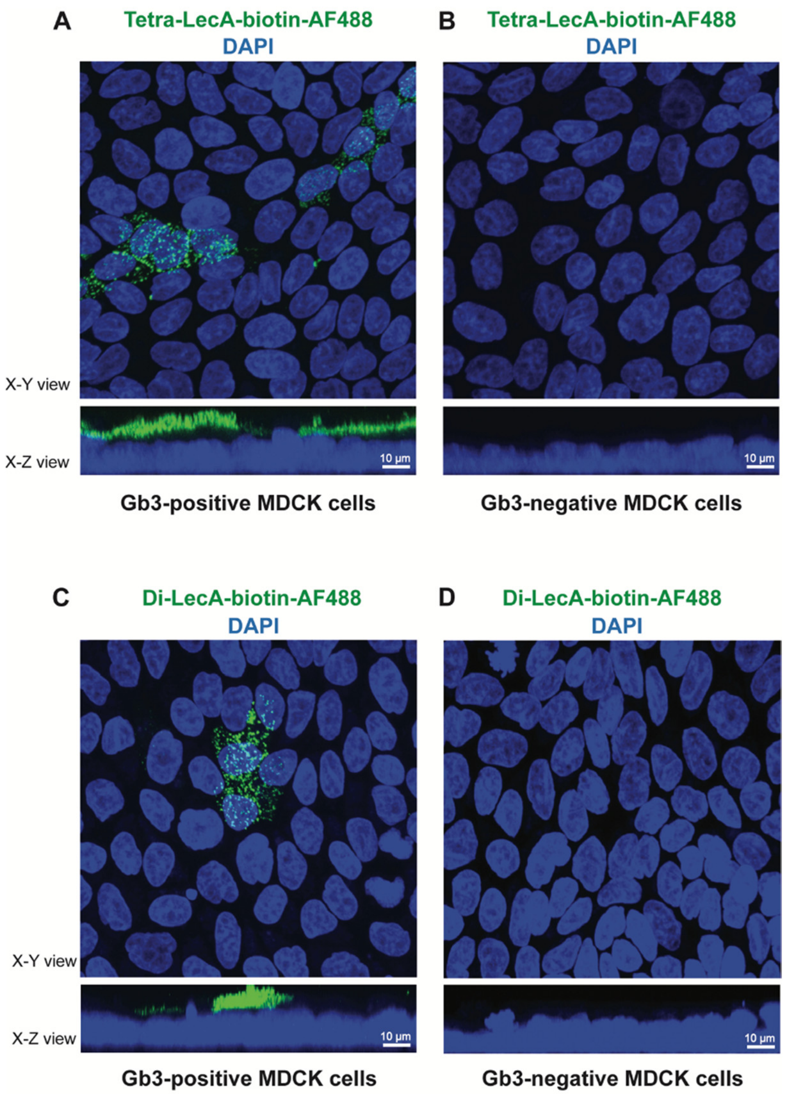

Figure S1. Binding studies of Tetra-LecA and Di-LecA to Gb3-positive and -negative MDCK cells. Gb3-positive and Gb3-negative (i.e., WT-) MDCK cells were seeded separately on Transwell filters for 4–5 days until the tight junctions (TJs) were well developed. Tetra-LecA-biotin-AF488 or Di-LecA-biotin-AF488 (100 nM) was pre-bound to the Ap cell surface for 30 min at 4 °C. Excess of lectins was removed by washing with ice-cold medium. After fixation and permeabilization, nuclei were stained with DAPI (blue), and images were acquired by confocal microscopy. Representative maximum intensity projections (X-Y view) and cross-sections of acquired z-stack (X-Z view) images are presented (scale bar: 10 µm). (A) Tetra-LecA-biotin-AF488 (green color) bound to Gb3-positive cells, and no binding to Gb3-negative MDCK cells was observed for the same acquisition parameters (B). (C) Di-LecA-biotin-AF488 (green color) bound to Gb3-positive MDCK cells. (D) No binding of Di-LecA-biotin-AF488 (green color) to Gb3-negative MDCK cells could be observed.

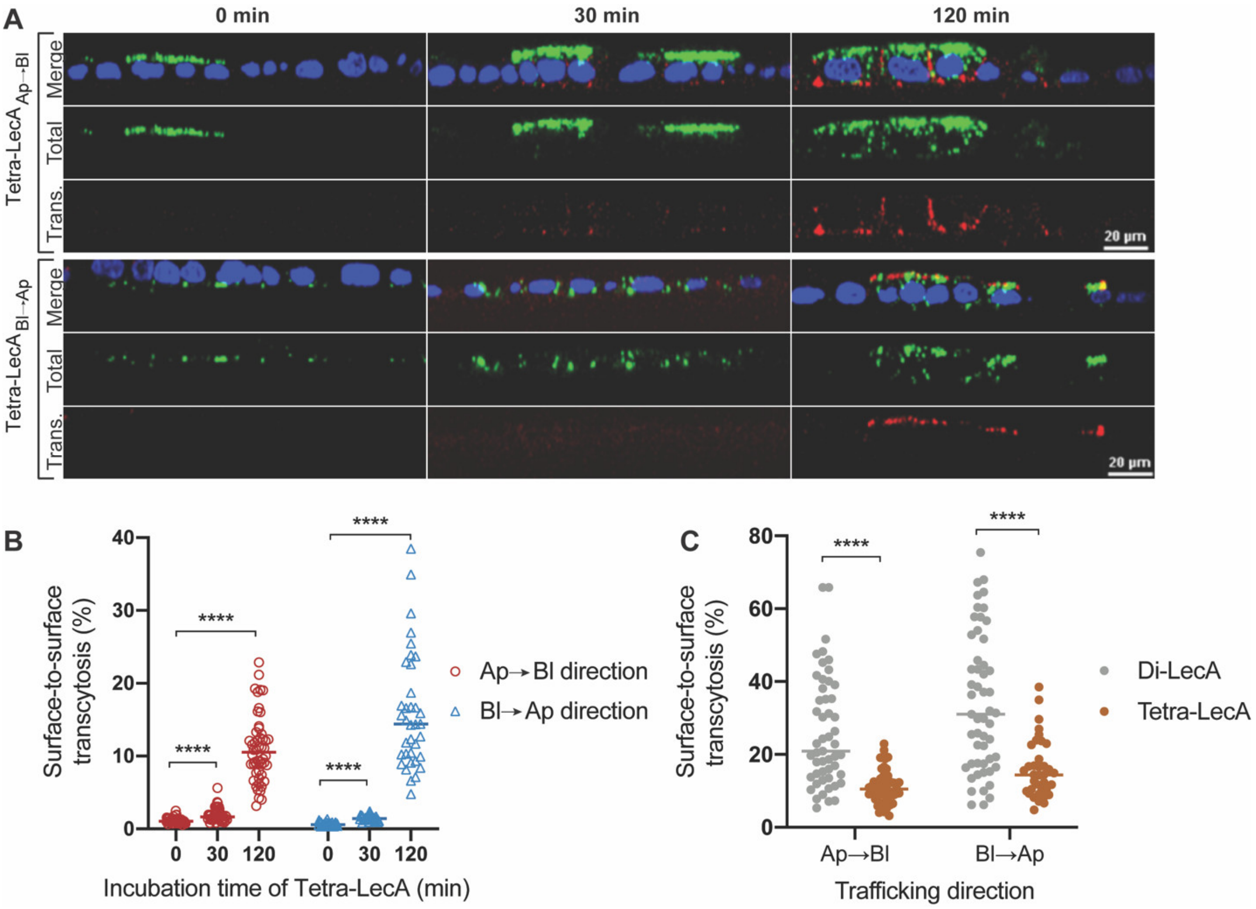

Figure S2. Surface-to-surface transcytosis of Tetra-LecA. (A) Transcytosed Tetra-LecA-biotin-AF488 was measured in Gb3-positive MDCK cells. Representative cross-sections of acquired z-stack images are shown. Arrival of Tetra-LecA-biotin-AF488 (green) was detected by using streptavidin-AF647 (red) from the opposite side after indicated incubation periods of transport in Ap→Bl and Bl→Ap directions (scale bar: 20 μm). (B) The surface-to-surface transcytosis efficiencies of Tetra-LecA from two directions of transport were quantified. For each time point, the included individual cell number n was greater or equal to 30. (C) The surface-to-surface transcytosis efficiencies of Di-LecA and Tetra-LecA after 120 min of incubation were compared. For each time point, the included individual cell number n was greater or equal to 30. The Mann-Whitney test was used to calculate the significant difference between each group. Mean values are shown. **** $P < 0.0001$.

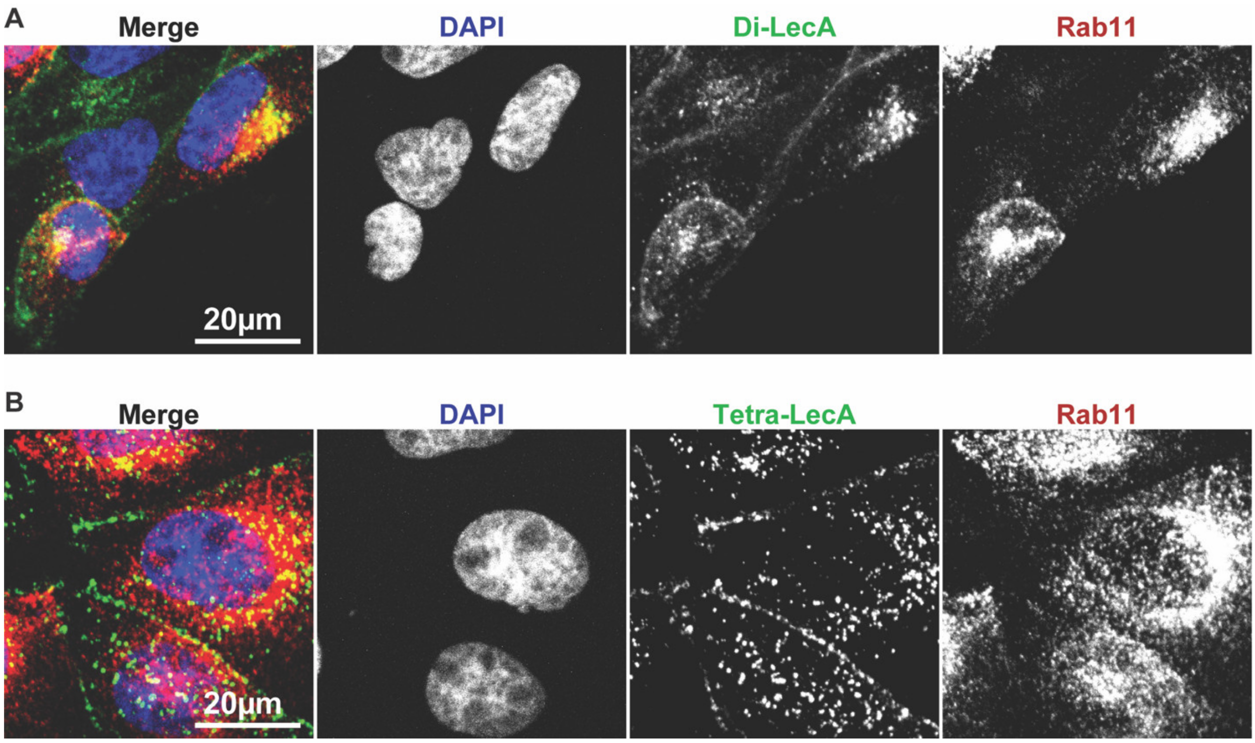

Figure S3. Co-localization analysis of Di-LecA and Tetra-LecA with the small GTPase protein Rab11. (A) Fluorescent Di-LecA (green) was pre-bound to non-polarized Gb3-positive MDCK cells at 4 °C for 30 min. Cells were washed with ice-cold cell culture medium and shifted to 37 °C for 30 min of incubation subsequently. After cell fixation and permeabilization, nuclei were stained with DAPI (blue), and the small GTPase protein Rab11, marker of the recycling endosomes, was stained with a primary antibody, followed by incubation with the corresponding secondary antibody (red). Images were acquired via confocal microscopy (scale bar: 20 µm). Di-LecA accumulated in the perinuclear area and largely colocalized with the small GTPase protein Rab11 after 30 min of trafficking. (B) Fluorescent Tetra-LecA (Green) was pre-bound to non-polarized Gb3-positive MDCK cells at 4 °C for 30 min and intracellular trafficking through the small GTPase protein Rab11 was analyzed. Similar experimental procedures were conducted as illustrated in A). Tetra-LecA colocalized with the small GTPase protein Rab11 after 30 min of transport in non-polarized Gb3-positive MDCK cells (scale bar: 20 µm).

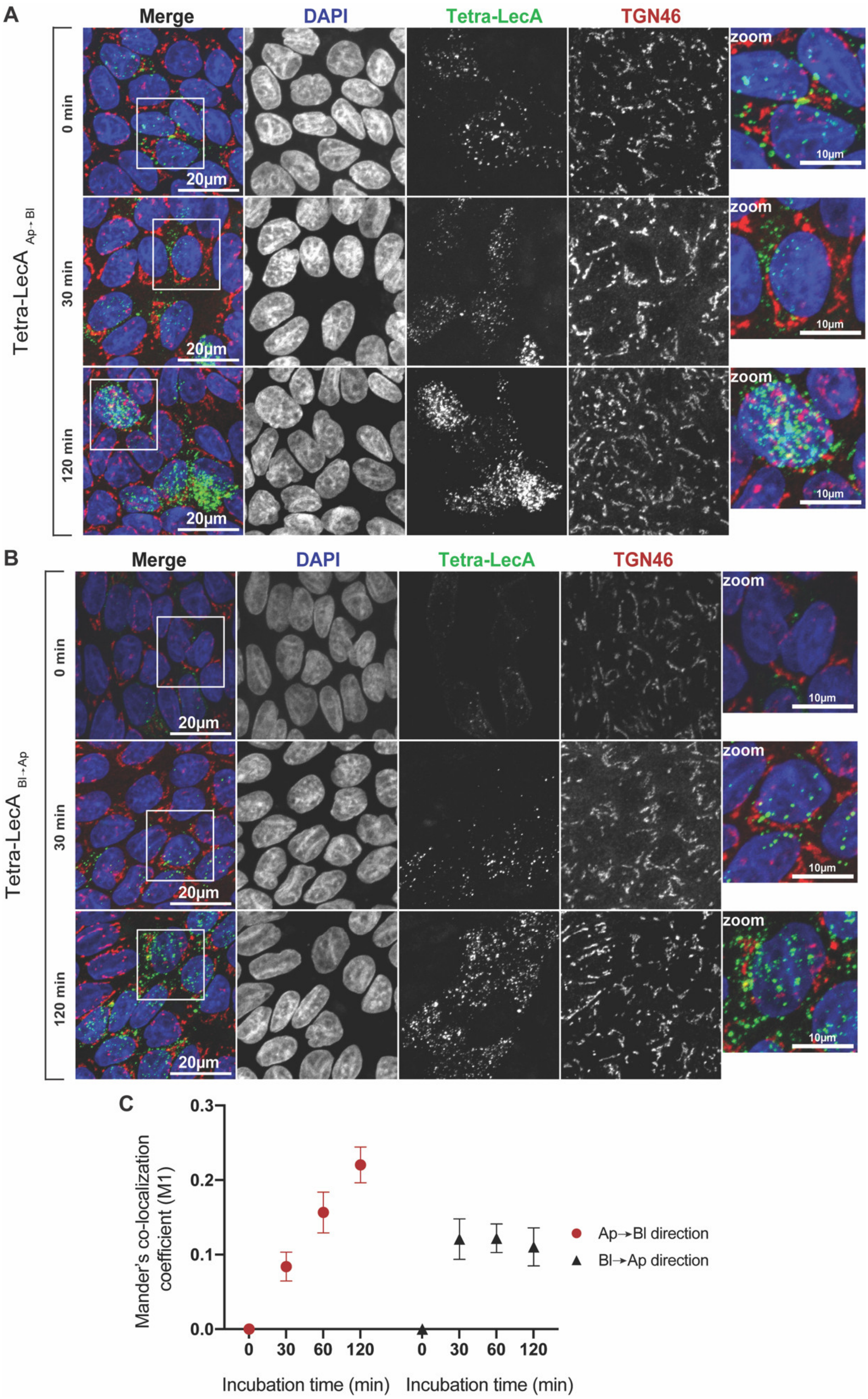

Figure S4. Co-localization analysis of Tetra-LecA with the *trans*-Golgi network marker TGN46. (A) Fluorescent Tetra-LecA (green) was pre-bound to the Ap membrane of Gb3-positive MDCK cells at 4 °C for 30 min. After washing out the excess of Tetra-LecA, cells were shifted to 37 °C for the indicated incubation periods, to allow endocytosis and intracellular trafficking from Ap to Bl side. After cell fixation and permeabilization, nuclei were stained with DAPI (blue), and TGN46 was

stained with a primary antibody, followed by incubation with the corresponding secondary antibody (red). Images were acquired via confocal microscopy, and representative maximum intensity projections are presented (scale bar: 20 µm; scale bar in the zoom: 10 µm). (B) Fluorescent Tetra-LecA was pre-bound to the Bl cell surface and intracellular trafficking from Bl to Ap side through the TGN was analyzed. Exactly the same experimental conditions were applied as described in A). (C) The Mander´s co-localization coefficient M1 between Tetra-LecA and TGN46 was calculated for different time points of transport in Ap→Bl and Bl→Ap directions. For each time point, the included individual cell number n was greater than or equal to 30. The error bars represent the SD.

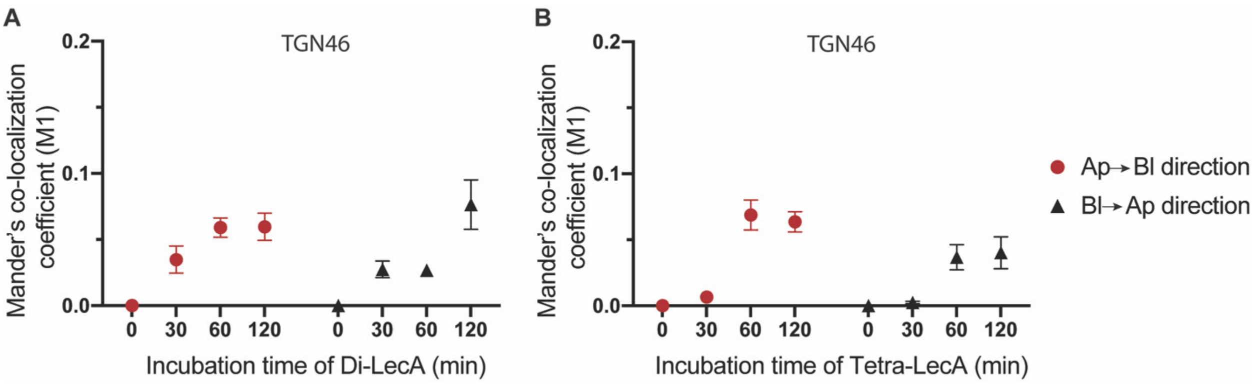

Figure S5. Co-localization analysis between Di-LecA or Tetra-LecA with TGN46 in Caco-2 cells. (A) The Mander's co-localization coefficient M1 between Di-LecA and TGN46 was quantified for different time points up to 2 h. Co-localization analysis showed that all coefficient values for different time points of incubation were below 0.1, suggesting a very low co-localization between Di-LecA and TGN46. (B) The Mander's co-localization coefficient M1 of Tetra-LecA with TGN46 for different time points was quantified. Low co-localization coefficient values (below 0.1) between Tetra-LecA and TGN46 were observed, suggesting a low co-localization. For each time point of trafficking, the involved cell number n was greater or equal to 30; the error bars represent the SD.

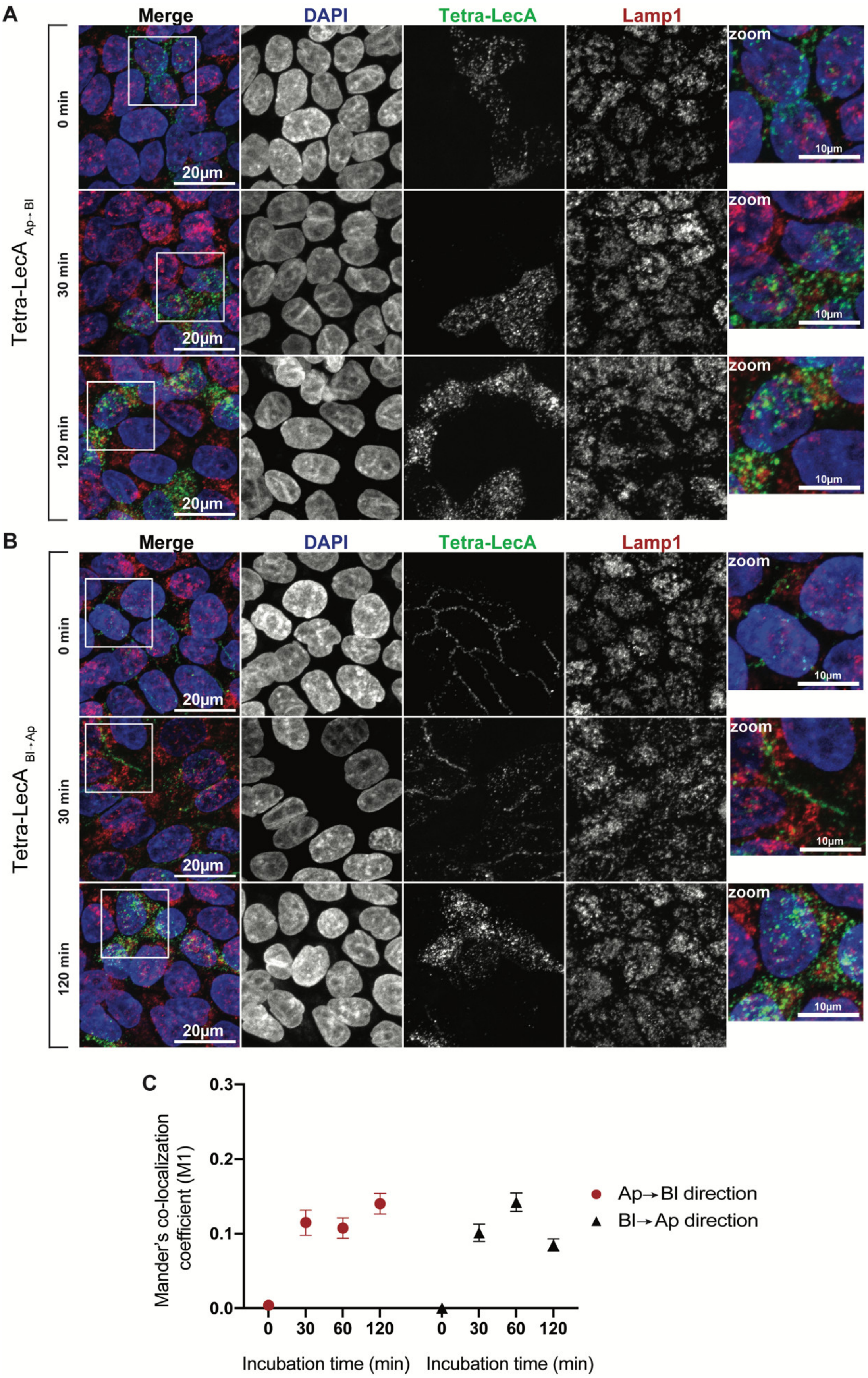

Figure S6. Co-localization analysis of Tetra-LecA with the lysosomal marker Lamp1. (A) Fluorescent Tetra-LecA (green) was added to the Ap surface of Gb3-positive MDCK cells at 4 °C for 30 min of incubation. After removing the unbound lectins, cells were shifted to 37 °C allowing trafficking processes from Ap to Bl membrane. After fixation and permeabilization, nuclei were stained with DAPI (blue). Simultaneously, Lamp1 was stained with a primary antibody, followed

by incubation with the corresponding secondary antibody (red). Images were acquired via confocal microscopy, and representative maximum intensity projections are presented (scale bar: 20 µm; scale bar in the zoom: 10 µm). (B) Fluorescent Tetra-LecA (green) was pre-bound to the Bl membrane of Gb3-positive MDCK cells at 4 °C for 30 min. Similar experimental procedures were conducted as illustrated in A). Images were acquired via confocal microscopy, and representative maximum intensity projections are presented (scale bar: 20 µm; scale bar in the zoom: 10 µm). (C) The Mander's co-localization coefficient M1 between Tetra-LecA and Lamp1 is presented for different time points of trafficking in Ap→Bl and Bl→Ap directions. For each time point, the involved cell number n was greater or equal to 30. The error bars represent the SD.

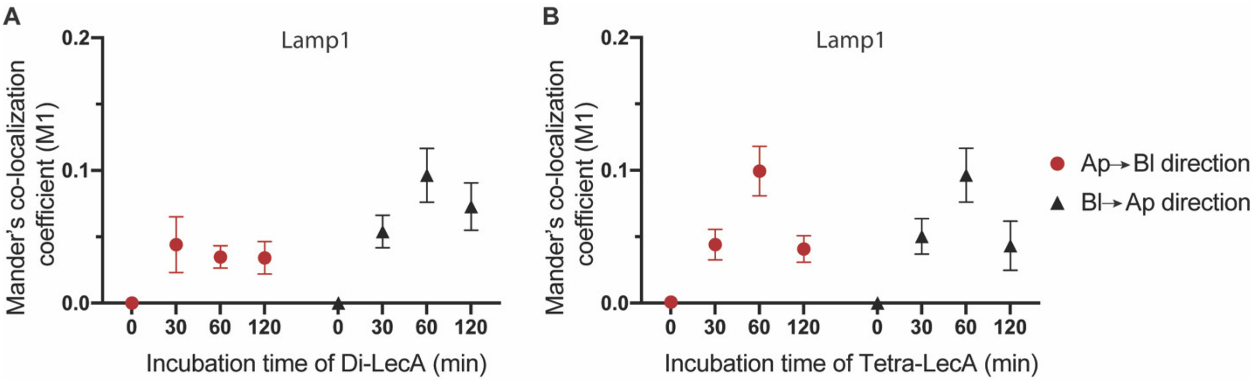

Figure S7. Co-localization analysis between Di-LecA or Tetra-LecA with Lamp1 in Caco-2 cells. (A) The Mander's co-localization coefficient M1 of Di-LecA with Lamp1 was quantified for different time points of transport up to 2 h in Ap→Bl and Bl→Ap directions. Low co-localization coefficient values (below 0.1) between Di-LecA and Lamp1 were observed. (B) The Mander's co-localization coefficient M1 between Tetra-LecA and Lamp1 for different time points was quantified. The degree of overlap between Tetra-LecA and Lamp1 was low (around 0.1), suggesting a low co-localization. For each time points, the involved cell number n was greater or equal to 30; the error bars represent the SD.

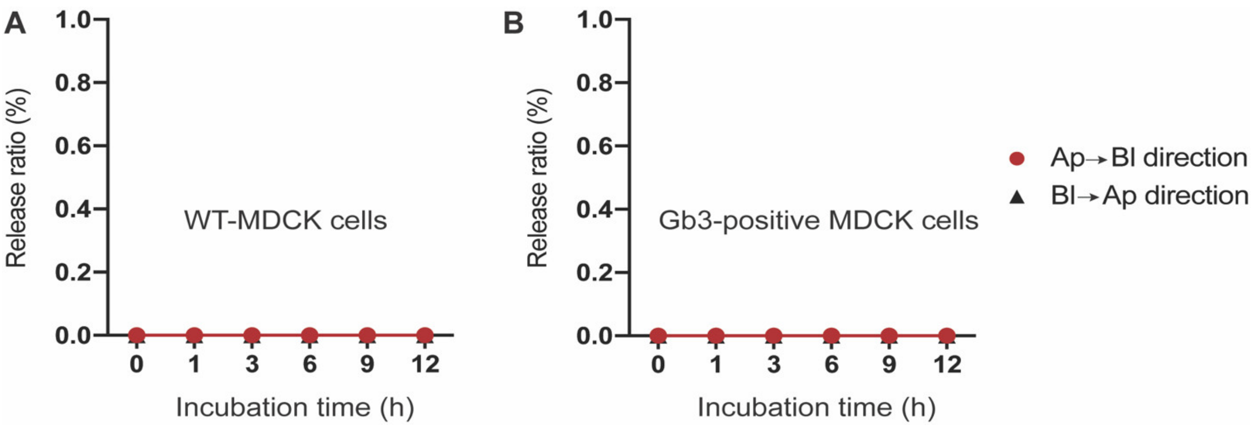

Figure S8. Release efficiency of dextran-FITC. (A) To confirm the impenetrability of used cell monolayers that consisted of WT-MDCK cells, dextran-FITC with a molecular size of 20 kDa was applied either on the top (Ap) or the bottom (Bl) of the chambers. After indicated time points of incubation, the cell culture medium was collected, and the fluorescence signal intensities of dextran-FITC were measured using a microplate reader. The release efficiencies of dextran-FITC were calculated according to the formula shown in Section 2.5. (B) The impermeability of used cell monolayers composed of Gb3-positive MDCK cells was evaluated by using dextran-FITC. The same experimental procedures were conducted as described in (A).

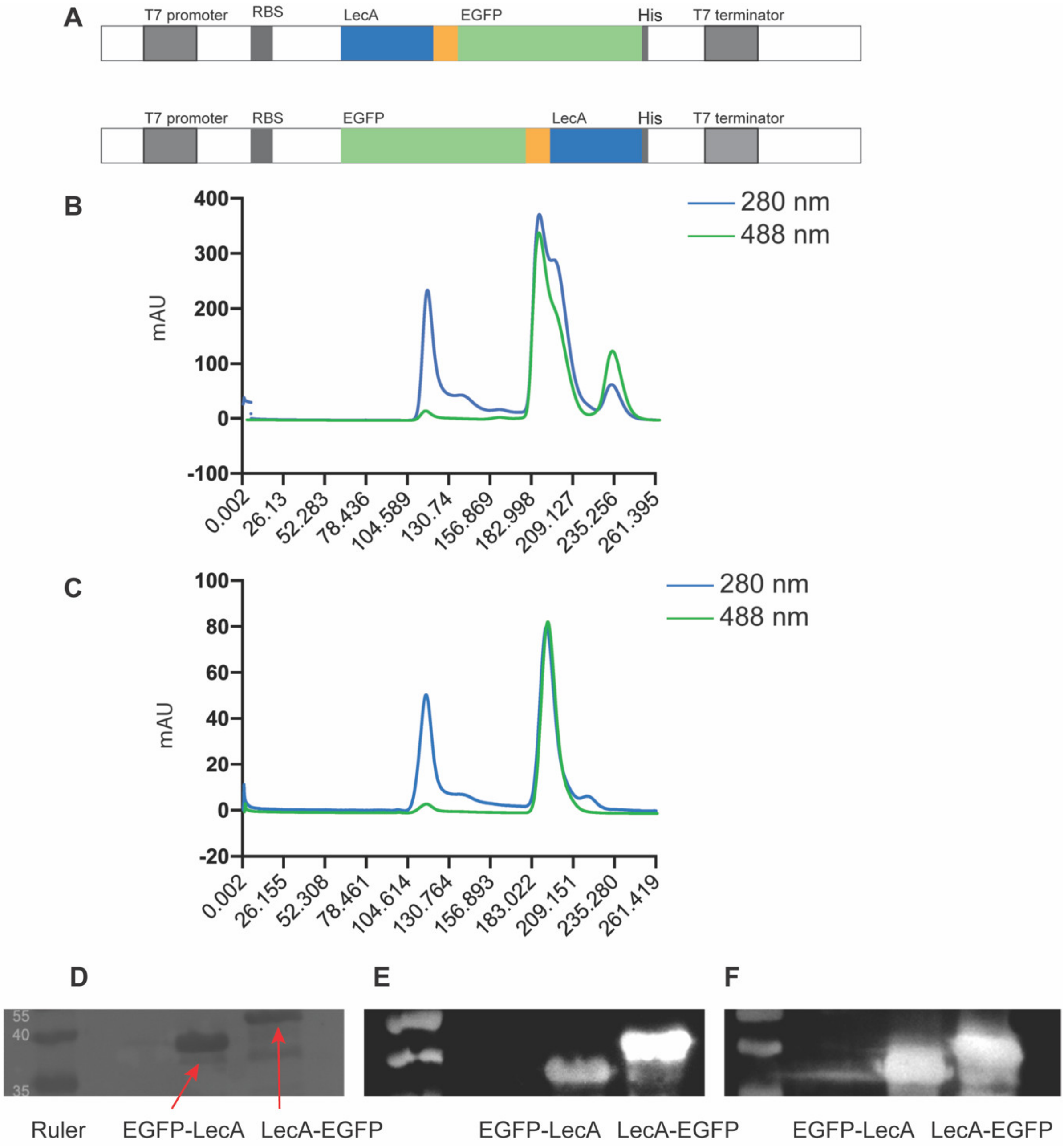

Figure S9. Production, purification, and validation of LecA-fusion proteins. (A) The genes of LecA chimeras were designed as illustrated in the scheme. Plasmids containing the genes of *LecA-EGFP* and *EGFP-LecA* were transformed separately into *E. coli* NiCo21 (DE3) cells, to produce corresponding proteins. A HisTrap column using a Ni-NTA agarose stationary phase was used as a first round of purification. Subsequently, samples were collected and prepared for size exclusion chromatography. LecA-EGFP (B) and EGFP-LecA (C) from the overlap fractions were harvested and collected for further experiments, respectively. (D) The molecular sizes of LecA-fusion proteins were confirmed via SDS-PAGE. Using Western blotting, the antibodies towards LecA and EGFP were applied to confirm whether LecA-fusion proteins contain the LecA (E) and EGFP fraction (F), respectively.

Molecular Dynamics simulations

In LecA, each monomer forms a small jelly-roll type β-sandwich fold, consisting of two curved sheets. Each β-sheet comprises four antiparallel β-strands. The tetramer formation occurs by interaction between the largest sheets for one interface and by contacts between C-terminal and N-terminal moieties for the other interface (Figure S10A) [64]. In accordance with the X-ray structure definition, the former refers to as dimer of A/B (C/D) type and is stabilized by hydrophobic interactions (Figure S10B). The other dimer is of A/C (B/D) type and stabilized mainly by hydrogen bonds between terminal residues of the neighboring monomers (Figure S10C).

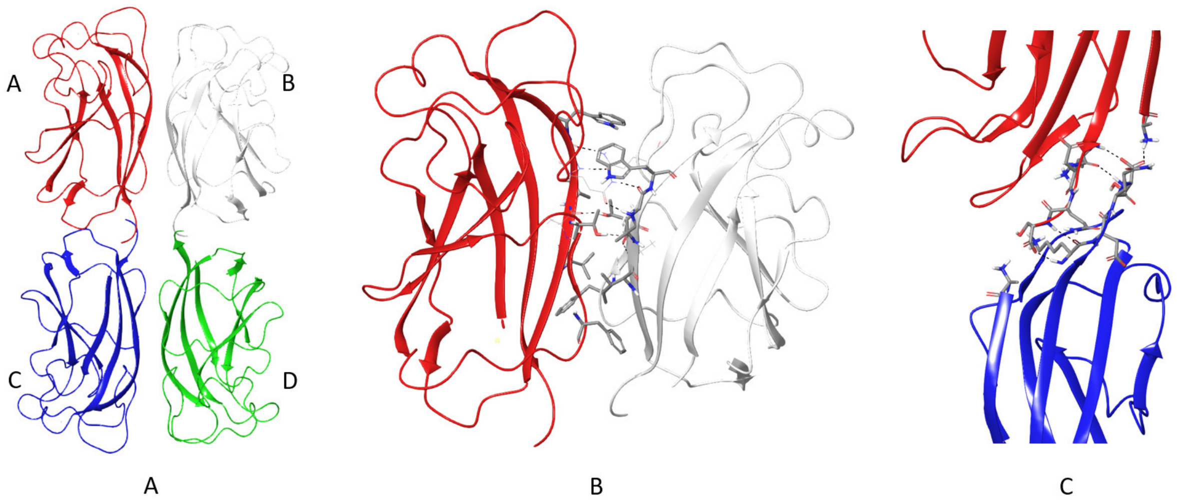

Figure S10. The structure of LecA tetramer (pdb code: 5D21) (A). The interactions stabilizing the A/B dimer type (B) and A/C dimer type (C) are shown in sticks.

In the context of current study, we hypothesized that LecA fusion monomer would have altered N- and C-terminal structure and dynamics, which would destabilize the A/C (B/D) contacts (Figure S10). As a result, the probability of the LecA tetramer formation would vary. At the current step of modelling, EGFP was excluded for the sake of computational efficiency. From the experimental constructs two protein sequences were derived: 1) LecA monomer bearing the linker sequence GGGS followed by the flag-tag sequence DYKDDDDK (referred to as 'connecting sequence', CS) and 6×His-tag (Figure S11A); 2) LecA monomer bearing the CS at the C-terminus (Figure S11B). In addition, the constructs of LecA monomer bearing only CS at the N-terminus (Figure S11C) and only a 6×His-tag at the C-terminus (Figure S11D) were considered to deduce their accumulative effect.

A) LecA-CS sequence:

AWKGEVLANNEAGQVTSIIYNPGDVITIVAAGWASYGPTQKWGPQGDRE-HPDQGLICHDAFCGALVMKIGNSGTIPVNTGLFRWVAPNNVQGAITLI-YNDVPGTYGNNSGSFSVNIGKDQSGGGSDYKDDDDK

B) CS-LecA-6×His-tag sequence:

GGGSDYKDDDDKAWKGEVLANNEAGQVTSIIYNPGDVITIVAAGWASY-GPTQKWGPQGDREHPDQGLICHDAFCGALVMKIGNSGTIPVNTGLFRWVAP-NNVQGAITLIYNDVPGTYGNNSGSFSVNIGKDQSLEHHHHHH

C) CS-LecA sequence:

GGGSDYKDDDDKAWKGEVLANNEAGQVTSIIYNPGDVITIVAAGWASY-GPTQKWGPQGDREHPDQGLICHDAFCGALVMKIGNSGTIPVNTGLFRWVAP-NNVQGAITLIYNDVPGTYGNNSGSFSVNIGKDQS

D) LecA-6×His-tag sequence:

AWKGEVLANNEAGQVTSIIYNPGDVITIVAAGWASYGPTQKWGPQGDRE-HPDQGLICHDAFCGALVMKIGNSGTIPVNTGLFRWVAPNNVQGAITLI-YNDVPGTYGNNSGSFSVNIGKDQSLEHHHHHH

Figure S11. Diverse sequences containing LecA used for accelerated molecular dynamics modelling.

To get a deeper insight into the structure and dynamics of the above-mentioned constructs, accelerated molecular dynamics simulations (AMD) were performed. The AMD approach has been chosen to enhance the sampling. Such an acceleration allowed to tackle protein folding, including helical structure formation [35,65] and beta structure formation [37].

Figure S12 depicts the secondary structure content and the probability of every residue to form a β-sheet or an α-helix along the AMD trajectories for all molecular systems studied. Our data shows the tendency of the 6×His-tag to form α-helix, which is in line with a previous modelling report [66]. According to the experimental data, the His-tag may influence the protein dynamics and stability [67]. The increase of protein B-factors was revealed by X-ray crystallography [68]. Two-dimensional infrared vibrational echo spectroscopy revealed the altered protein motions on a short (picosecond) and long (>100ps) time scale [69]. The incorporation of His-tag may also affect the protein oligomerization [70]. The structural notion of this alteration is still unclear and the effect is rather case-dependent. Nonetheless, these effects become crucial for such a protein derivatization as in synthetic chimeras.

The helix of the His-tag is populated along the whole AMD trajectory of 1 microsecond, which due to the enhanced sampling covered a longer timeframe when benchmarked against conventional MD. The helix formation is along with the intactness of the overall LecA folding, which is illustrated by permanent β-structure content similar to that in LecA without any tags. Starting from the beginning of the trajectory, the His-tag forms an α-helix, along with unordered conformation (Figure S12A). The overall probability of the His-tag to form a helix amounted to 20% (Figure S12B). This probability decreased with the serial number of the residue in the sequence, so that the terminal histidine had the lowest probability to form a helix (Figure S13).

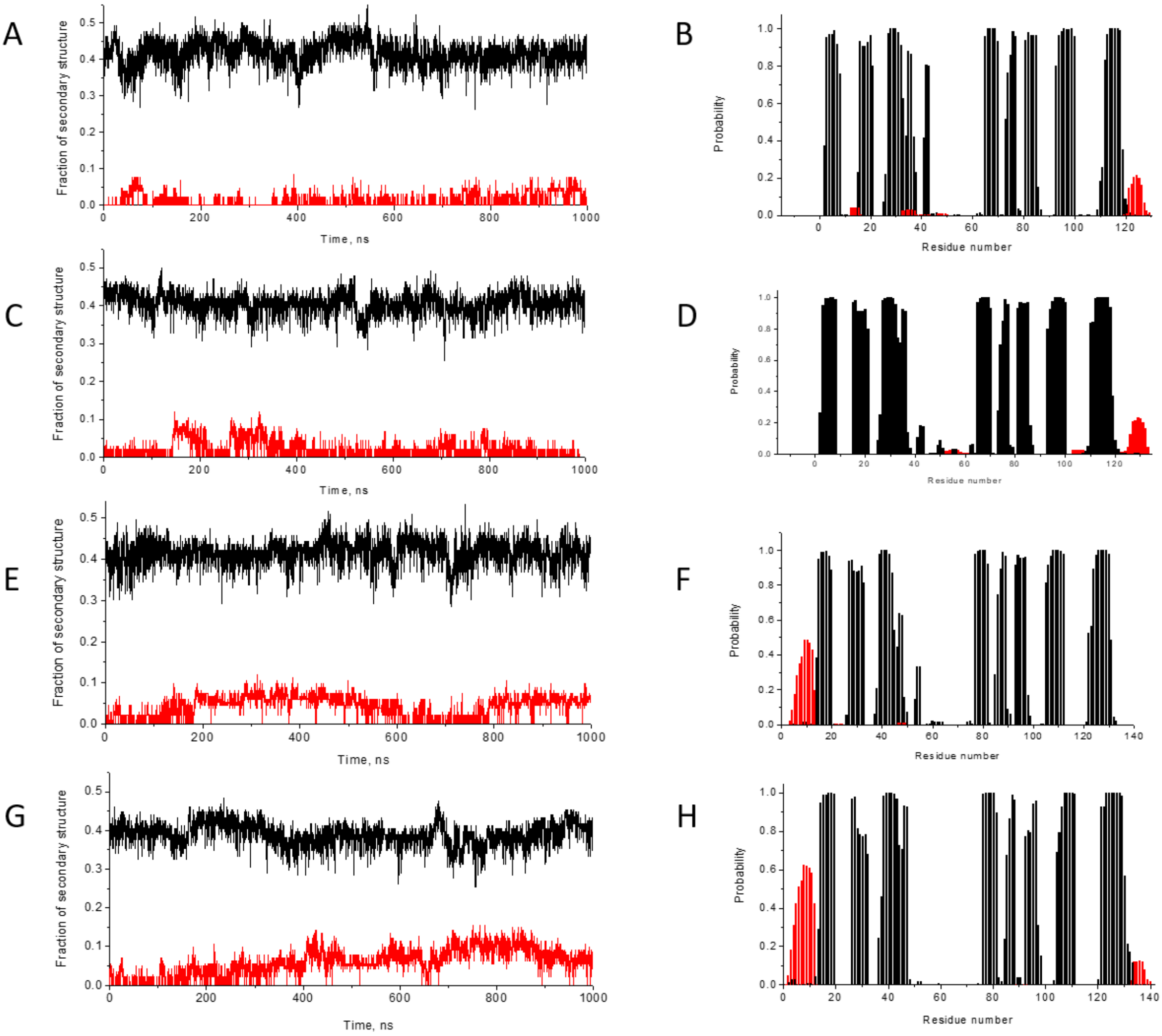

Figure S12. Secondary structure content in LecA-6×His-tag (A-B), LecA-CS (C-D), CS-LecA (E-F), CS-LecA-6×His-tag (G-H). The overall content of β-sheets and α-helices along the trajectory of four systems (A, C, E, G) and the probability of each amino acid to contribute to these structures, calculated along the trajectory as a function of residue number (B, D, F, H).

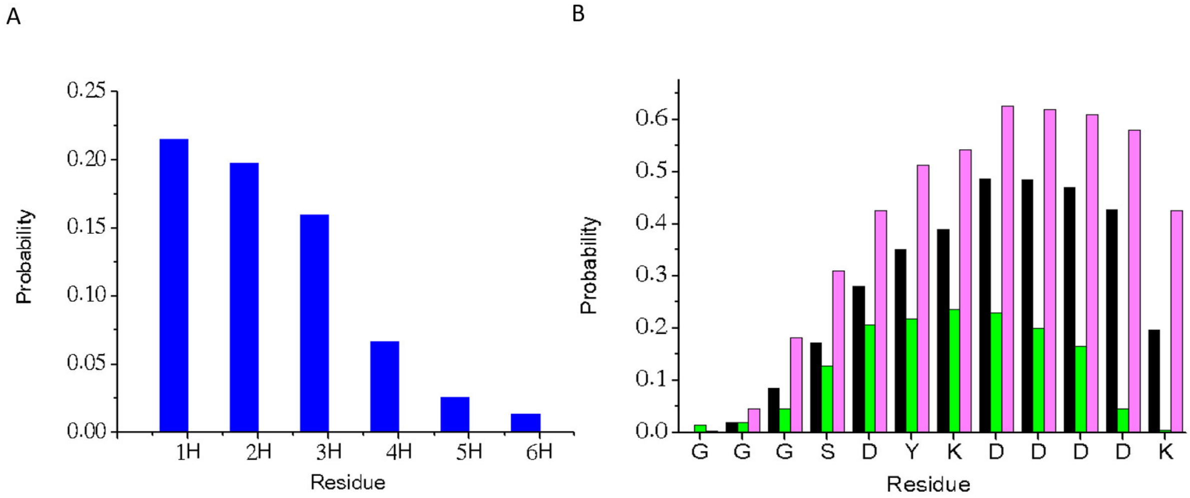

Figure S13. The probability of each residue to form a helix along the AMD trajectory for the 6×His (A) in LecA-6×His-tag and for CS (B) in systems: LecA-CS – green, CS-LecA – black, CS-LecA-6×His-tag – pink.

The CS is prone to form an α-helix with a probability higher than for the 6×His-tag. It varies from 25% to 50% depending on the CS position in respect to LecA. In LecA-CS, the probability is around 25% (Figure S12C-D, Figure S13B). In CS-LecA, the probability increases up to 50% (Figure S12E-F, Figure S13B). The probability of each residue to form a helix depends on the type of the construct. In LecA-CS, the distribution of the probability to form a helix versus the residue serial number resembles the bell-shaped curve. From this, the sequence of three glycine residues, linked directly to LecA, is excluded since it has a minimal probability to form a helix. In CS-LecA, the probability of residues attached to LecA to form a helix notably increases. Furthermore, when CS-LecA is enriched by the 6×His-tag at the C-terminus, the probability of CS to form a helix increases up to 65% (Figure S12G-H, Figure S13B).

To unravel the molecular reasons of the influence of the 6×His-tag on the additional CS stabilization in helical conformation, we further performed the analysis of the dihedral angles principal component (DPCA) analysis. The protein free energy landscape (FEL) is represented as a function of principal component eigenvectors. The free energy profile of LecA-CS dynamics projected along two the first principal components PC1 and PC2 reveals one broad global energy minimum and some local energy basins (Figure S14). The basins are separated by energy barriers of 2.5-3 kcal/mol. The basins are represented by random conformations of CS with a variety of its orientation in respect to the protein. In contrast, the main basin is represented by a cluster of structures characterized by the helical geometry of CS.

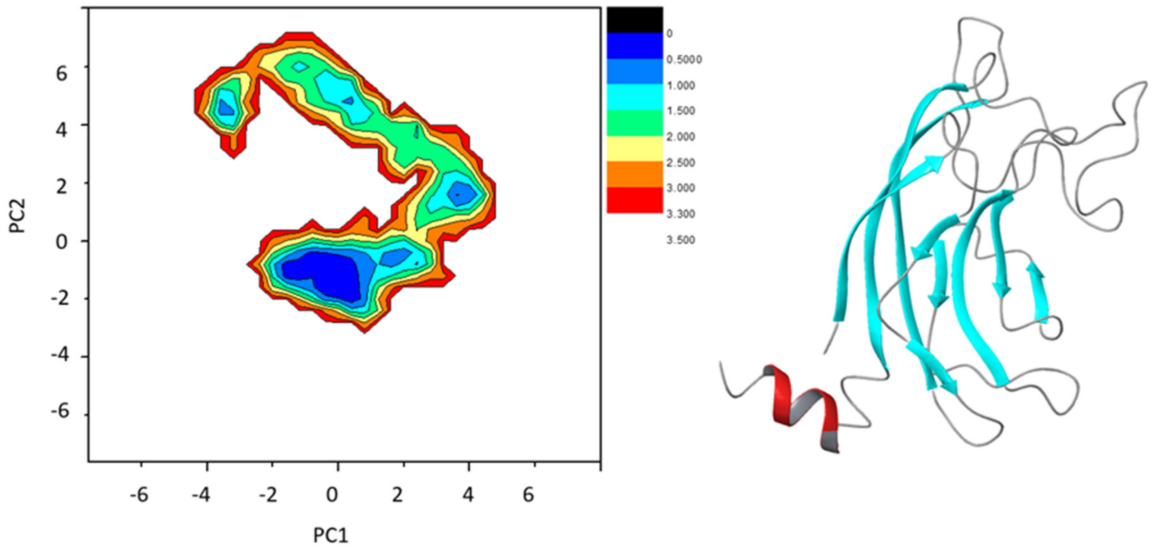

Figure S14. DPCA analysis of an AMD trajectory of LecA-CS and the representative structure of the most populated state.

The FEL map of CS-LecA also demonstrates one global minimum and a number of local minima. The main basin is represented by the helical conformation CS, predominantly oriented as it is shown in Figure S15.

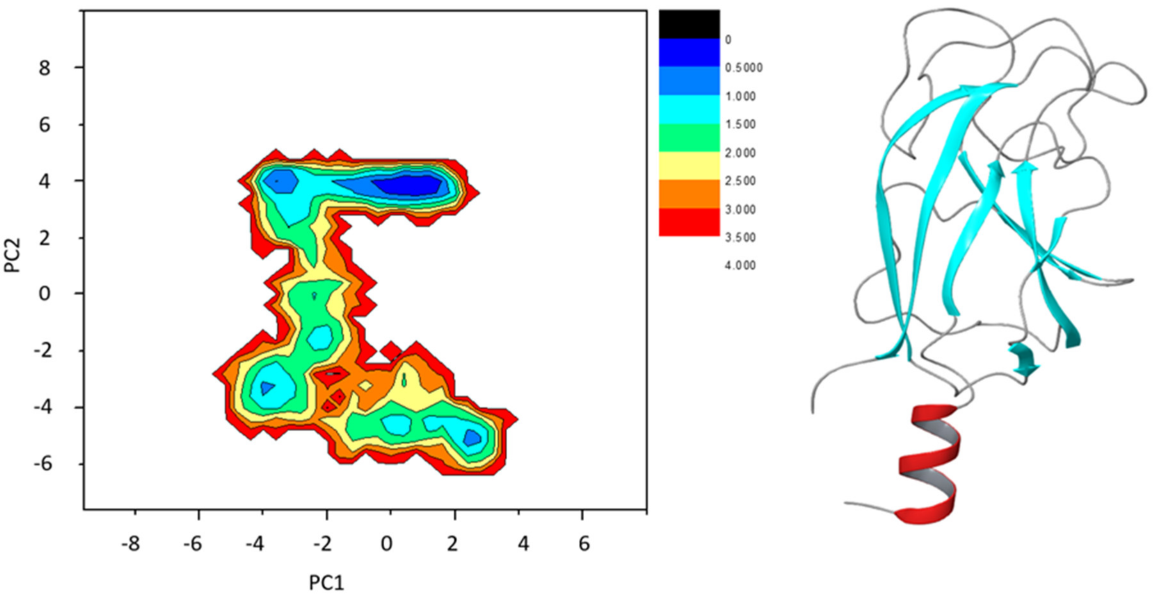

Figure S15. DPCA analysis of an AMD trajectory of CS-LecA and the representative structure of the most populated state.

The analysis of CS-LecA-6×His-tag shows that the increase of the helix formation probability by the linker of up to 65% is due to the contacts between the CS and 6×His-tag. The most populated state, up to 50%, is characterized by the CS helix and the unordered state of 6×His-tag (Figure S16 A), a less populated cluster is represented by two helices formed by both CS and His-tag interacting with each other (Figure S16 B). The least populated cluster is represented by the structure when the CS and 6×His-tag interact in a mode of β-strands (Figure S16 C).

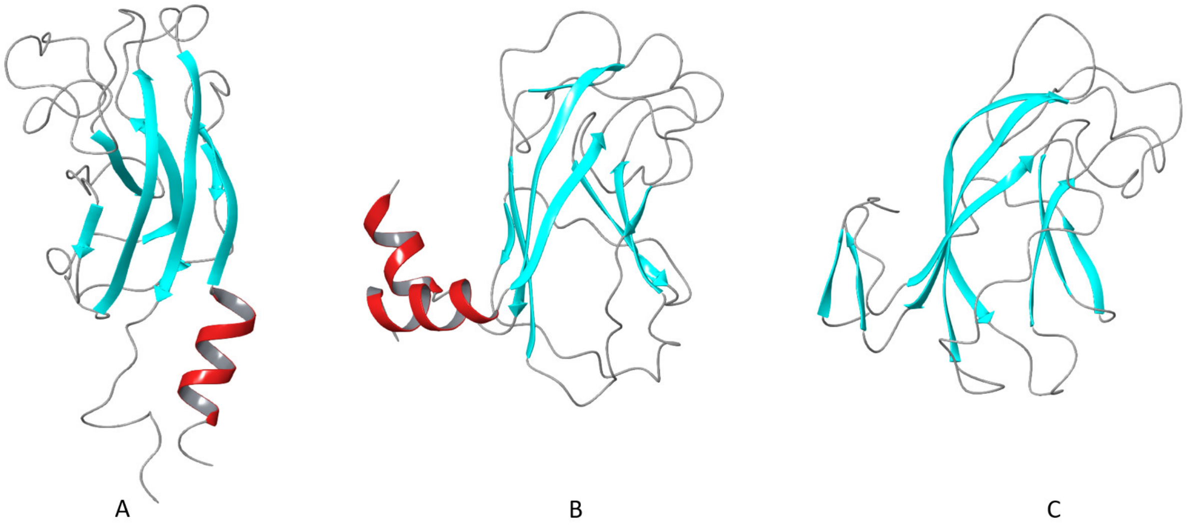

Figure S16. The representative structures of three most populated states from DPCA analysis of an AMD trajectory of LecA-CS.

Summing up, the analysis of evolution of the CS in the course of AMD trajectories revealed the tendency of the CS to form a helix. The probability of that is higher when

the CS is attached to the N-terminus of LecA. The presence of a 6×His-tag at the C-terminus gives an additional stabilization to the CS linked to the N-terminus. This stabilization occurs due to the interactions between the CS and the 6×His-tag. The increase of conformational rigidity in the fragment CS-LecA-6×His-tag can reduce the probability of the formation of a native-like tetramer due to the disruption of contacts in the A/C (B/D) dimers. Extrapolating this to LecA and EGFP fusion proteins, the rigid CS located at the N-terminus of LecA and breaking contacts of A/C (B/D) type can result in a dimeric fusion protein where LecA molecules interact as in A/B (C/D) dimers. A less rigid CS located at the C-terminus of LecA appears to have less influence on the organization of the tetrameric fusion protein. This is in line with our experimental findings that LecA-EGFP cross-linked two membrane surfaces whereas EGFP-LecA binding to the membranes revealed no cross-linking.
